# Aberrant striatal firing mediates impulsive decision-making in a mouse model of Parkinson's disease

**DOI:** 10.1093/brain/awaf312

**Published:** 2025-09-04

**Authors:** Xiaowen Zhuang, Julia Lemak, Sadhana Sridhar, Alexandra B Nelson

**Affiliations:** Kavli Institute for Fundamental Neuroscience, UCSF, San Francisco, CA 94158, USA; Weill Institute for Neurosciences, UCSF, San Francisco, CA 94158, USA; Department of Neurology, UCSF, San Francisco, CA 94158, USA; Aligning Science Across Parkinson’s (ASAP) Collaborative Research Network, Chevy Chase, MD 20815, USA; Kavli Institute for Fundamental Neuroscience, UCSF, San Francisco, CA 94158, USA; Weill Institute for Neurosciences, UCSF, San Francisco, CA 94158, USA; Department of Neurology, UCSF, San Francisco, CA 94158, USA; Aligning Science Across Parkinson’s (ASAP) Collaborative Research Network, Chevy Chase, MD 20815, USA; Kavli Institute for Fundamental Neuroscience, UCSF, San Francisco, CA 94158, USA; Weill Institute for Neurosciences, UCSF, San Francisco, CA 94158, USA; Department of Neurology, UCSF, San Francisco, CA 94158, USA; Aligning Science Across Parkinson’s (ASAP) Collaborative Research Network, Chevy Chase, MD 20815, USA; Kavli Institute for Fundamental Neuroscience, UCSF, San Francisco, CA 94158, USA; Weill Institute for Neurosciences, UCSF, San Francisco, CA 94158, USA; Department of Neurology, UCSF, San Francisco, CA 94158, USA; Aligning Science Across Parkinson’s (ASAP) Collaborative Research Network, Chevy Chase, MD 20815, USA

**Keywords:** dopamine agonist, pramipexole, delay discounting, basal ganglia, striatum, medium spiny neurons

## Abstract

Parkinson's disease (PD) is characterized by progressive neurodegeneration, which is associated with motor and non-motor symptoms. Dopamine replacement therapy can remediate motor symptoms, but can also cause impulse control disorder (ICD), characterized by pathological gambling, hypersexuality and/or compulsive shopping. Approximately 14%–40% of all medicated PD patients suffer from ICD. Despite the high prevalence of ICD in medicated PD patients, we know little of its mechanisms, and the main therapeutic strategy is reducing or eliminating dopamine agonist medication. Human imaging studies suggest that the input nucleus of the basal ganglia, the striatum, may be a critical site of circuit dysfunction in ICD.

To explore the cellular and circuit mechanisms of ICD, we developed a mouse model in which we administered the dopamine D2/3 agonist pramipexole to parkinsonian and healthy control mice. ICD-like behaviour was assessed using a delay discounting task. Delay discounting is a normal cognitive phenomenon, in which the value of a reward decreases according to the time needed to wait for it. Impulsivity is measured as the preference for immediate (small) over delayed (large) rewards. We combined this mouse model with chemogenetics and *in vivo* optically identified single-unit recordings to examine how dopamine agonists act on vulnerable striatal circuitry to mediate impulsive decision-making.

We found that in parkinsonian mice, therapeutic doses of dopamine D2/3 receptor (D2/3R) or D1 receptor (D1R) agonists drove more pronounced delay discounting, reminiscent of what has been reported in PD/ICD patients on medication. In contrast, healthy mice did not become more impulsive when given the same dose of dopamine agonist. The clinically relevant dopamine D2/3R agonist pramipexole induced marked bidirectional changes in the firing of striatal direct and indirect pathway neurons in parkinsonian mice. Chronic pramipexole treatment potentiated these changes in striatal physiology and decision-making behaviour. Furthermore, chemogenetic excitation of direct pathway striatal neurons or inhibition of indirect pathway neurons induced impulsive decision-making in the absence of dopamine agonists. These findings indicate that abnormal striatal activity plays a causal role in mediating ICD-like behaviours. Together, they provide a robust mouse model and insights into ICD pathophysiology.

## Teaser

Little is known about the mechanisms underlying impulse control disorder in Parkinson's disease. Zhuang *et al.* show that in parkinsonian mice, the dopamine D2/3R agonist pramipexole increases delay discounting, mirroring effects seen in patients, with the changes driven by abnormal activity in the striatum.

## Introduction

We often weigh immediate versus distant costs and benefits in making decisions. Impulsive decision-making is characterized by intolerance for long-term costs and preference for more immediate rewards. Impulsivity is seen in a number of neuropsychiatric conditions, including neurodegenerative disorders, psychiatric disease and drug addiction.^1,2^ One notable example is impulse control disorder (ICD), a complication of Parkinson's disease (PD) treatment. PD is characterized by progressive degeneration of midbrain dopamine neurons, which contributes to motor impairment, such as slowing of movement (bradykinesia), tremor and rigidity.^3^ Dopamine replacement therapy, particularly with D2/3-type receptor (D2/3R) agonists, alleviates motor deficits, but can be complicated by the development of ICD. In response to dopamine agonists, up to 40% of PD patients develop non-motor symptoms like pathological gambling, binge eating or hypersexuality; this cognitive-behavioural syndrome is termed ICD.^4,5^ Our current understanding of ICD is primarily informed by epidemiological and imaging studies in clinical populations. There are few studies in animal models, and the pathophysiological mechanisms remain unknown.^6-9^

The cognitive profile of PD/ICD provides a few clues as to its origins. Those with ICD show a preference for immediate rewards, and an intolerance for delays. This phenomenon has been studied by measuring delay discounting, a normal cognitive phenomenon in which the value of a reward decreases according to the time needed to wait for it. People with ICD tend to choose immediate but small rewards over delayed/large rewards in delay discounting tasks.^10-12^ In healthy animals, prior studies suggest that delay discounting depends on the frontal cortex, striatum (caudate and putamen) and dopamine.^13-15^ In healthy non-human primates, dopamine agonist infusion in the striatum induced impulsive choices during a delay discounting task.^16^ In addition, striatal activity encodes key variables in delay discounting behaviour.^17-19^

One of the most distinctive features of ICD in PD is its relationship to dopamine agonist medication. Dose reduction or discontinuation typically eliminate symptoms of ICD, suggesting dopamine signalling may reversibly modulate striatal circuits to drive ICD.^20^ Within the striatum, dopamine regulates striatal projection neurons, medium spiny neurons (MSNs). Direct pathway MSNs (dMSNs) express the dopamine D1 receptor (D1R) and indirect pathway MSNs (iMSNs) express the D2 receptor (D2R).^21^ Striatal dopamine release is hypothesized to excite dMSNs and inhibit iMSNs, based on *ex vivo* and *in vivo* recordings.^22-26^ Indeed, in a mouse model of PD, treatment with the dopamine precursor levodopa, or dopamine agonists, causes acute bidirectional changes in dMSN and iMSN activity.^25,27^ Another complication of dopamine replacement therapy, levodopa-induced dyskinesia, is associated with high dMSN activity and low iMSN activity.^25,28,29^ While the neural correlates of ICD are unknown, one possibility is that chronic dopamine depletion in PD triggers circuit adaptations, which in turn create circuit-level vulnerability to the effects of dopamine agonists, including an imbalance in dMSN and iMSN activity driving ICD.

To explore this hypothesis, and the cellular and circuit mechanisms of ICD, we created a mouse model of PD/ICD. In mildly parkinsonian (but not in healthy) mice, dopamine D2/3 or D1 agonists led to altered delay discounting behaviour reminiscent of those seen in PD/ICD.^4,5^ In exploring the neural correlates, we found that the D2/3 agonist pramipexole (PPX) induced marked bidirectional changes in dMSN and iMSN firing in parkinsonian mice. Chronic PPX treatment further potentiated these changes in striatal physiology and decision-making behaviour. Chemogenetic inhibition of iMSNs or excitation of dMSNs in the DMS drove impulsive decision-making. Taken together, our findings provide a robust mouse model of ICD, and shed light on how dopamine agonists may induce pathological impulsivity in PD.

## Materials and methods

### Animals

All mice were bred on a C57BL/6 background and housed under a 12-h light/dark cycle with *ad libitum* access to food and water unless stated otherwise. All experiments were performed during the light phase. We used male and female mice aged 3–7 months old. Littermates of the same sex were randomly assigned to experimental groups. All experiments were conducted with the approval of the Institutional Animal Care and Use Committee at the University of California, San Francisco, and complied with local and national ethical and legal regulations regarding the use of mice in research.

### Surgical procedures

A detailed surgery protocol can be found at doi:10.17504/protocols.io.b9kxr4xn. To produce parkinsonian or control mice, the bilateral dorsolateral striatum (DLS) (+0.8 mm anterior-posterior, ±2.0–2.2 mm medial-lateral, −2.5 mm dorsal-ventral) were injected using a 33-gauge cannula (Plastics One) with 6-hydroxydopamine (6-OHDA)-bromide (1.5 μl per site, 2.5 μg/μl) or sterile saline, respectively. To minimize uptake of the toxin by noradrenergic axons, desipramine (Sigma-Aldrich, 25 mg/kg intraperitoneal) was administered immediately prior to surgery. Details regarding viral injections and procedures for *in vivo* recordings are provided in the Supplementary material.

### Motor assessment

Details of the accelerating rotarod and open field tests can be found at doi:10.17504/protocols.io.q26g7yo4kgwz/v1. Briefly, motor function was evaluated using the accelerating rotarod test (Ugo Basile) at two time points: 3 weeks after 6-OHDA injection surgery (Pre) and 4 h post-injection of PPX or saline. To minimize the effect of motor learning on rotarod performance, each mouse underwent no more than two sessions: one before and one after pharmacological administration. Details of the rotarod protocol and additional open field procedures are provided in the Supplementary material.

### Operant training and assessment

A detailed protocol for operant training can be found at doi:10.17504/protocols.io.4r3l22k93l1y/v2. Other details about the operant training phases, as well as the associated scripts, are included in the Supplementary material. Operant training began around 3 weeks after 6-OHDA (or saline) injection using a three-phase shaping protocol. Each mouse was trained in the same operant chamber throughout the study. Male and female mice were trained in separate operant chambers. To promote consistency, testing was performed at the same time of day, 5 days a week.

### *In vivo* electrophysiology

A detailed protocol for *in vivo* electrophysiology can be found at doi:10.17504/protocols.io.b9ucr6sw. Briefly, 1 week after the optrode array implantation, mice were habituated to tethering and the open field chamber for at least 2 days. After habituation, experimental sessions occurred at least once per week for 4–6 weeks. Further details on recording parameters, spike sorting and classification of single units are provided in the Supplementary material.

### Pharmacology

Details are available at doi:10.17504/protocols.io.e6nvw1nbzlmk/v2. Details on all other drug preparations, dosages and administration protocols are provided in the Supplementary material.

### Histology, quantification and microscopy

A detailed protocol for the preparation of histological sections can be found at doi:10.17504/protocols.io.b9ubr6sn. For details on quantitative histology, see the Supplementary material. After behavioural, *in vivo* electrophysiology and chemogenetic experiments, mice were deeply anaesthetized with intraperitoneal ketamine-xylazine and transcardially perfused with 4% paraformaldehyde in PBS. Electrode locations were marked by electrolytic lesioning, and post-mortem verification of dopamine depletion and viral expression was performed by tyrosine hydroxylase immunostaining, with animals lacking either excluded from analysis. Details are provided in the Supplementary material.

### Statistical analysis

The experimental design and statistical analysis of all key experiments are summarized in Table S1 of the Supplementary material. This table includes statistical tests, N (animals), n (units or cells), *P*-values and the associated figures. Power calculations were performed based on pilot data and previous studies. We aimed to achieve >80% power to detect a significant difference with a two-sided α of 0.05, using the statistical tests reported in Table S1. All data are presented as the mean ± standard error of the mean (SEM). Statistical tests were performed using GraphPad Prism 10. In all analyses, a *P*-value of <0.05 was considered statistically significant, unless Bonferroni corrected for multiple comparisons, in which case the *P*-value was multiplied by the number of comparisons. For complete experimental design, see the Supplementary material.

## Results

### Dopamine D2/3R agonist pramipexole causes impulsive decision-making in parkinsonian mice

**Figure 1:**
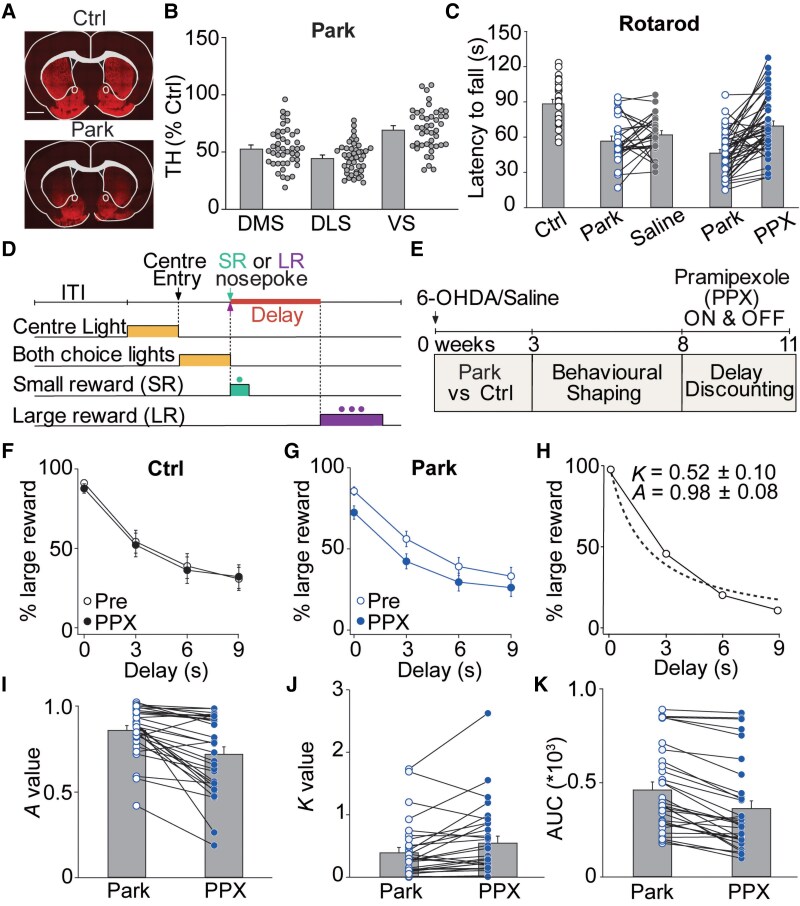
**Dopamine D2/3B agonist pramipexole causes impulsive decision-making in parkinsonian mice.** (**A** and **B**) Post-mortem coronal sections containing the striatum were immunostained for tyrosine hydroxylase (TH, red). (**A**) Representative striatal sections from saline-injected (*top*) and 6-OHDA-injected (*bottom*) mice. (**B**) Average TH immunofluorescence across different anterior striatal subregions in parkinsonian (Park) mice, normalized to saline-injected control (Ctrl) sections (*n* = 44, DMS versus DLS: *P* = 0.09; VS versus DMS: *P* = 0.0003; VS versus DLS: *P* < 0.0001). (**C**) On the accelerating rotarod test, parkinsonian mice showed a lower latency to fall, but improved with pramipexole (PPX) (0.5 mg/kg) (Ctrl: *n* = 31, Park/PPX: *n* = 45, Park/Saline: *n* = 27; Ctrl versus Park/PPX: *P* < 0.001, Park versus PPX: *P* < 0.001, Park versus Saline: *P* = 0.95). (**D**) Delay discounting task structure. Each delay was tested in a separate block, up to 9 s. ITI = intertrial interval. (**E**) Experimental timeline. (**F** and **G**) Percentage of trials in which mice chose the delayed/large reward across delays during baseline (open circles) and following PPX treatment (filled circles). PPX did not significantly change decision-making in healthy control mice (**F**, *n* = 16; *P* > 0.05 at all delays), but reduced the likelihood of delayed/large choices at every delay in parkinsonian mice (**G**, *n* = 31; 0 s: *P* < 0.0001, 3 s: *P* < 0.0001, 6 s: *P* < 0.0001, 9 s: *P* < 0.0087). Ctrl/Pre versus Park/Pre: *P* > 0.05 at all delays. (**H**) Hyperbolic discounting function, fitted (dashed line) to representative delay discounting behaviour in one mouse. The intercept and steepness of the curve were quantified by *A* and *K*, respectively. (**I**–**K**). *A*, *K* and area under the curve (AUC) values associated with delay discounting before (Park) and following PPX treatments (PPX) in parkinsonian mice (*n* = 31, **I**, *P* < 0.0001; **J**, *P* = 0.03; **K**, *P* < 0.0001). All data are presented as mean ± standard error of the mean. Scale bar = 1 mm. 6-OHDA = 6-hydroxydopamine; DMS = dorsomedial striatum; DLS = dorsolateral striatum; VS = ventral striatum. See also Supplementary Figs 1 and 2.

To model early-stage PD, for which dopamine agonist therapy is often used,^30^ we injected a low dose of the dopaminergic neurotoxin 6-OHDA bilaterally in the dorsolateral striatum (DLS). We used saline-injected mice as controls. 6-OHDA resulted in partial loss of midbrain dopamine neurons, with greater impact on axons in the dorsal striatum (Fig. 1A). Using tyrosine hydroxylase (TH) as a surrogate marker for dopamine neurons, we found approximately 50% loss of TH signal in the rostral dorsolateral and dorsomedial striatum (DMS), with less marked depletion in the ventral striatum (VS; Fig. 1B and Supplementary Fig. 1A and B; see statistics in figure legend and details in the Supplementary material and Supplementary Table 1). There were fewer dopaminergic cell bodies in the substantia nigra pars compacta (SNc) in 6-OHDA-treated versus control mice (Supplementary Fig. 1C and D). 6-OHDA-treated mice showed mild motor impairment on the accelerating rotarod test (Fig. 1C), consistent with a mild-moderate parkinsonian phenotype. As in people with early-stage PD, motor performance was remediated by dopamine replacement therapy. Treatment with the dopamine D2/3-type agonist, PPX (0.5 mg/kg), improved rotarod performance (Fig. 1C). Consistent with findings in healthy rodents,^31^ PPX caused an acute reduction in movement in both control and parkinsonian mice. However, increased locomotor activity was seen at later time points in parkinsonian mice, consistent with a therapeutic response (Supplementary Fig. 1E and F). These findings indicate the bilateral/partial 6-OHDA model shows key behavioural features of early-stage PD, which are responsive to dopamine agonist medication.

To model ICD-related alterations in decision-making, we took advantage of a normal cognitive phenomenon, delay discounting, in which the value of a reward is discounted by the time needed to wait for it.^32^ Delay discounting behaviour is abnormal in individuals with ICD, with more pronounced discounting, or intolerance for delays.^10-12^ We adapted a rodent delay discounting task for use in healthy and parkinsonian mice (Fig. 1D). Prior to training in the delay discounting task, control and parkinsonian mice underwent behavioural shaping, with two phases of instrumental learning (Fig. 1E and Supplementary Fig. 1G and J). Parkinsonian mice showed slightly slower response latencies and learning rates during the first phase (Supplementary Fig. 1H and I), but eventually achieved similar performance in the second phase (Supplementary Fig. 1K and L). These results indicate that while the bilateral/partial 6-OHDA model shows mild motor deficits, it does not impair the fundamental capacity for instrumental learning.

In the delay discounting task, animals chose between two alternatives: an immediate, small reward, and a larger reward at delays of 0, 3, 6 or 9 s. During the task, both control and parkinsonian mice showed classic discounting behaviour, choosing the large reward less often as the associated delay increased (Fig. 1F and G). PPX-naive control and parkinsonian mice showed similar delay discounting. However, PPX (0.5 mg/kg) reduced the likelihood of delayed/large reward choices as compared with baseline in parkinsonian mice (Fig. 1G), similar to PD patients with ICD.^10-12^ Notably, delay discounting was not altered by PPX in control mice (Fig. 1F), consistent with the lower risk of ICD in people without PD.^33^ We found that male and female mice exhibited similar impulsivity after PPX treatment (Supplementary Fig. 2A–C). Together, these findings suggest that like people with PD/ICD, parkinsonian mice are more vulnerable to the effects of PPX on impulsive decision-making.

To determine whether PPX altered discounting behaviour indirectly through changes in motivation or attention, we monitored the number of omitted trials and response latencies. Mice showed low omission rates during baseline and PPX sessions (Supplementary Fig. 2D and E). As predicted from prior studies,^34,35^ choice latencies for the delayed/large and immediate/small reward increased and decreased, respectively, across delays, until reaching a similar level (Supplementary Fig. 2F and G). In parkinsonian mice treated with PPX, the latency to choose small/immediate rewards was shorter than at baseline, and modulation in response latencies according to outcome was absent (Supplementary Fig. 2H), suggesting PPX-induced impairment in goal-directed responding. These observations suggest that PPX does not impair overall motivation, and may indeed promote more vigorous pursuit of immediate small rewards.

To characterize impulsive decision-making in parkinsonian mice treated with PPX, we fitted a hyperbolic discounting function V = 100 × *A* / (1 + *K*D) to each mouse's delay discounting curve (Fig. 1H). This function can be used to quantify aspects of discounting behaviour.^32,36,37^ The probability of choosing a large reward (V) is devalued by the length of delay (D), scaled by the discounting propensity (*A* and *K*). *K* reflects sensitivity to delays, or an index of the discount rate (steepness of the curve); and parameter *A* reflects sensitivity to reward magnitude (intercept with the *y*-axis).^38,39^ PPX had variable effects on *A* and *K* in individual parkinsonian mice, but overall led to a decrease in *A* and increase in *K* (Fig. 1I and J). We also quantified the shape of the discounting curve by measuring area under the curve (AUC); in this analysis, a decrease in AUC indicated an increase in impulsive choice.^15,40^ PPX treatment led to a significant reduction in AUC in parkinsonian mice, suggesting that PPX shifted choices towards immediate/small rewards at all delays (Fig. 1K). Interestingly, in sessions following a 48 h PPX washout period, the *A* value recovered to baseline values, while differences in *K* and AUC persisted (Supplementary Fig. S2I–K). These results suggest that in parkinsonian mice, PPX acutely reduces sensitivity to differences in reward magnitude, and chronically enhances sensitivity to delays. Altogether, these findings demonstrate rodents can closely recapitulate key features of PD with ICD.

### Dopamine D1R agonist A77636 causes impulsive decision-making in parkinsonian mice

**Figure 2:**
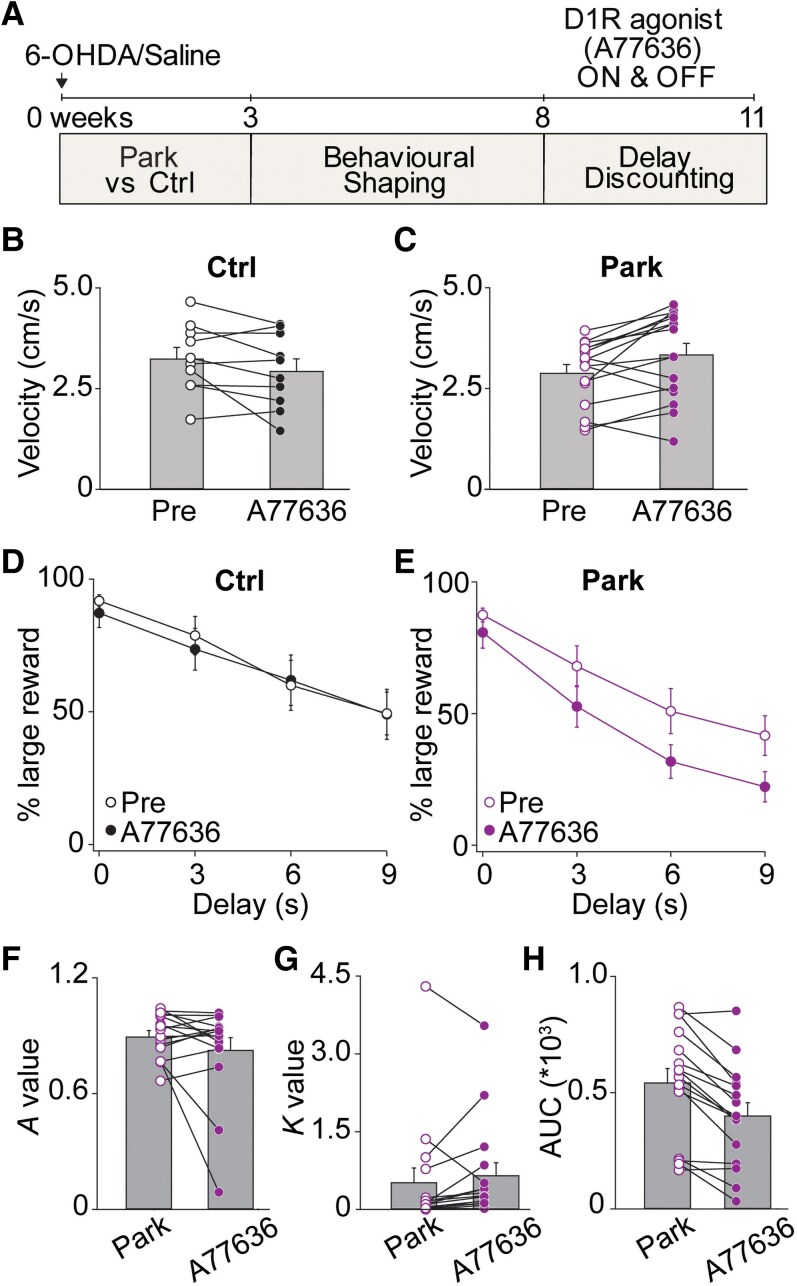
**Dopamine D1R agonist A77636 causes impulsive decision-making in parkinsonian mice.** (**A**) Experimental timeline. (**B** and **C**) Open field locomotor velocity in control (*left*) and parkinsonian (Park, *right*) mice following A77636 injection (Ctrl: *n* = 10, Park: *n* = 16; Ctrl: Pre versus A77636, *P* = 0.16, Park: Pre versus A77636, *P* = 0.02). (**D** and **E**) Percentage of trials in which mice chose the delayed/large reward across delays during baseline (open circles) and following A77636 injections (filled circles). A77636 did not significantly change decision-making in healthy control mice (**D**, *n* = 10; *P* > 0.05 at all delays), but reduced the likelihood of delayed/large choices at every delay in parkinsonian mice (**E**, *n* = 16; 0 s: *P* = 0.57, 3 s: *P* = 0.0047, 6 s: *P* = 0.0003, 9 s: *P* = 0.0002). Ctrl/Pre versus Park/Pre: *P* > 0.05 at all delays. (**F**–**H**). *A*, *K* and area under the curve (AUC) values associated with delay discounting during before (Park) and following A77636 treatments in parkinsonian mice (*n* = 16, **F**, *P* = 0.31; **G**, *P* = 0.0053; **H**, *P* < 0.0008). All data presented as means ± SEMs. See also Supplementary Fig. 3.

Studies of impulsivity in PD have largely focused on the role of dopamine D2/3R, likely due to the higher risk of ICD seen with D2/3 agonists over the dopamine precursor levodopa,^41^ and the fact that D1Rs agonists are not in clinical use. However, D1R agonists exhibit antiparkinsonian and reinforcing properties in rodents and primates.^42-44^ To address whether D1R agonists induce impulsive decision-making, we tested the long-acting D1R agonist, A77636, in parkinsonian mice performing the delay discounting task (Fig. 2A). At a dose of 1 mg/kg, A77636 increased locomotor speed in parkinsonian but not healthy mice (Fig. 2B and C). In a new cohort of mice, healthy and parkinsonian mice showed similar baseline delay discounting, but A77636 shifted the discounting curve in parkinsonian mice towards immediate/small rewards (Fig. 2D and E). Notably, A77636 had no significant effects on *A* in parkinsonian mice, but led to a significant increase in *K* (Fig. 2F and G), suggesting that parkinsonian mice treated with A77636 had greater difficulty tolerating delays. A77636 also reduced the AUC, suggesting an increase in impulsive decision-making (Fig. 2H). Similarly to our observations with PPX, the differences in *K* and AUC persisted even after a 48 h A77636 washout period (Supplementary Fig. 3F–H). Consistent with the original cohort, healthy and parkinsonian mice showed few omissions, and choice latency was modulated by reward size, suggesting that A77636 did not reduce task engagement or motivation (Supplementary Fig. 3A–D). Like PPX, A77636 led to a loss of outcome-dependent modulation of choice latency (Supplementary Fig. 3E). Together, these findings suggest that activation of D1R signalling can reproduce many of the effects of D2/3R agonist treatment on delay discounting behaviour.

### Changes in sensitivity to reward size relate to dopaminergic lesions in the striatum

**Figure 3:**
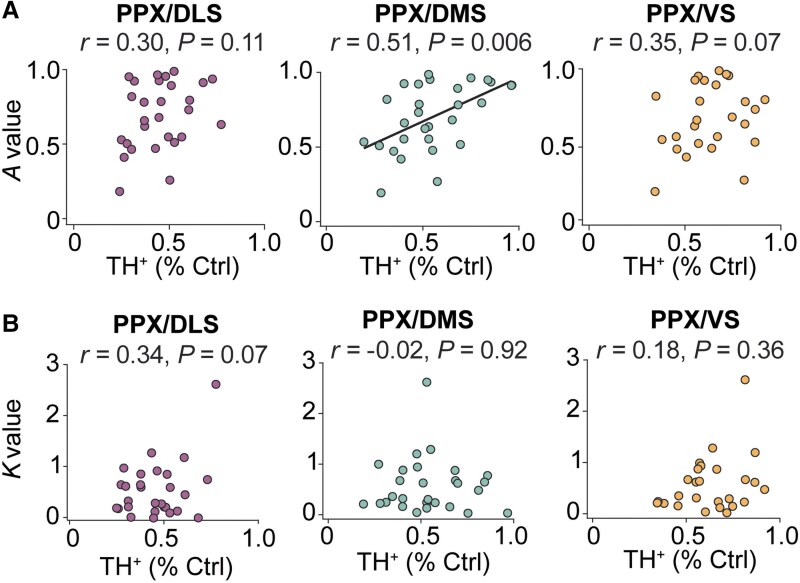
**Changes in sensitivity to reward size relate to dopaminergic lesions in the striatum.** (**A**) Scatter plots and fitted line demonstrating the correlation between the *A* values on parmipexole (PPX) and residual TH^+^ fluorescence intensity in the subregions of striatum in parkinsonian mice (*n* = 28 animals). (**B**) Scatter plot demonstrating the correlation between *K* values on PPX and residual TH^+^ fluorescence intensity in the subregions of striatum in parkinsonian mice (*n* = 28 animals). *Left*: Dorsolateral striatum (DLS); *middle*: dorsomedial striatum (DMS); *right*: ventral striatum (VS). See also Supplementary Fig. 4.

Vulnerability to ICD differs across individuals.^41^ Though the brain regions and neural mechanisms underlying this vulnerability are unclear, neuroimaging suggests that striatal volume and functional connectivity are risk factors.^45-47^ Those with ICD have longer PD symptom duration, suggesting that disease progression may contribute.^9^ To test whether differences in the severity of dopamine depletion predicted vulnerability to ICD in our mouse model, we correlated post-mortem measures of dopaminergic axonal integrity with key quantitative measures (*K* and *A*) of delay discounting (Fig. 3). We found that in parkinsonian mice treated with PPX, *A* values were positively correlated with residual TH immunofluorescence in the DMS, but not in the DLS or VS (Fig. 3A). *K* values did not correlate with TH immunofluorescence, whether in DMS, DLS or VS (Fig. 3B). A77636-induced impulsivity did not correlate with dopaminergic lesions in any subregions of the striatum (Supplementary Fig. 4). Taken together, these findings indicate that disease severity in the DMS is predictive of impaired reward magnitude sensitivity, one contributor to impulsivity.

### Pramipexole triggers bidirectional changes in striatal activity in parkinsonian mice

**Figure 4:**
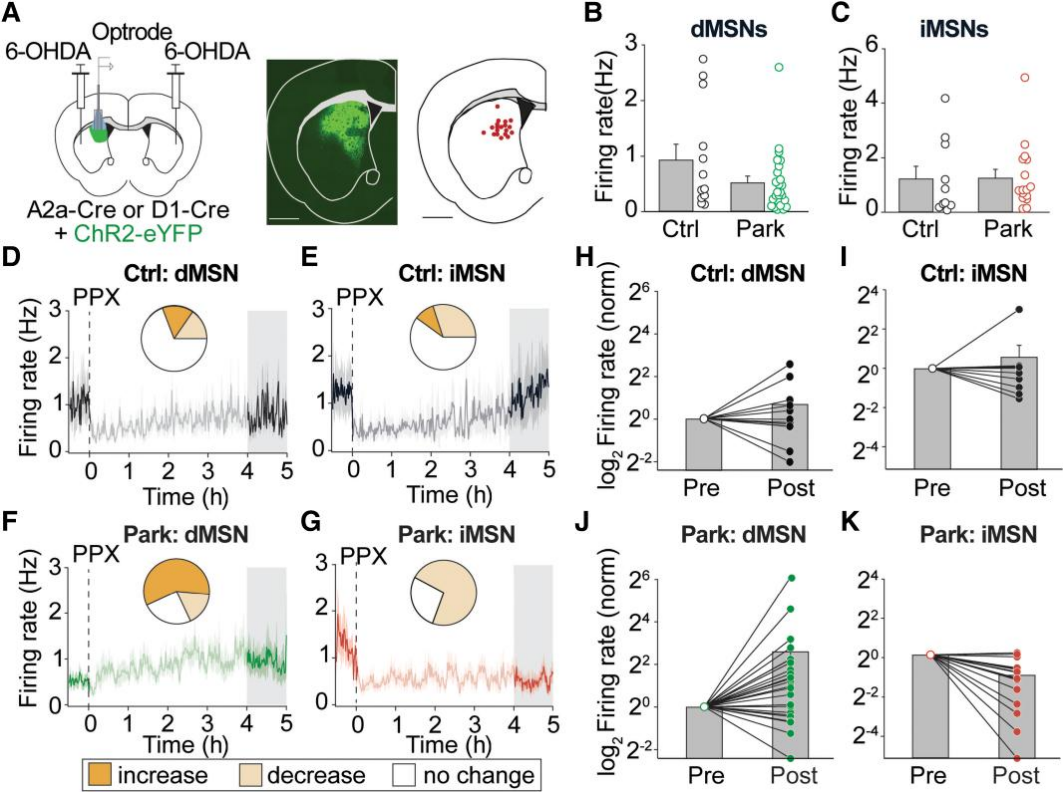
**Pramipexole triggers bidirectional changes in striatal activity in parkinsonian mice.** (**A**) *Left*: Schematic showing injection and optrode array implantation sites. *Middle*: Post-mortem histology confirming the expression of ChR2-eYFP (green). *Right*: Recording sites verified by electrolytic lesions. (**B** and **C**) Average baseline firing rates of optogenetically labelled dMSNs (**B**) and iMSNs (**C**) in healthy and parkinsonian mice [**B**, Ctrl: (*n* = 3 animals, *n* = 12 cells) versus Park: (*n* = 5 animals, *n* = 24 cells), *P* = 0.17; **C**, Ctrl: (*n* = 4 animals, *n* = 10 cells) versus Park: (*n* = 4 animals, *n* = 16 cells), *P* = 0.70]. (**D**–**G**) The effect of pramipexole (PPX) on optogenetically labelled dMSNs (**D** and **F**) and iMSNs (**E** and **G**). The shaded area at 4–5 h post-injection represents the time of all behavioural experiments; firing rates were compared between baseline and this period [**D**: (*n* = 3 animals, *n* = 12 cells), *P* = 0.91; **E**: (*n* = 4 animals, *n* = 10 cells), *P* = 0.38; **F**: (*n*= 5 animals, *n* = 24 cells), *P* = 0.01; **G**: (*n* = 4 animals, *n* = 16 cells), *P* < 0.001]. *Insets*: The proportion of optogenetically identified dMSNs and iMSNs whose firing rate increased, decreased or had no response to PPX (**D**: increase: 15.4%, decrease: 15.4%, no change: 69.2%; **E**: increase: 10.0%, decrease: 30.0%, no change: 60.0%; **F**: increase: 58.3%, decrease: 16.7%, no change: 25.0%, *P* = 0.01; **G**: increase: 0%, decrease: 72.7%, no change: 27.3%, *P* = 0.002). (**H**–**K**) Summary of normalized response of dMSN (**H**) and iMSN (**I**) firing rates to PPX (compared with baseline) in healthy and parkinsonian mice (same data as displayed in **D**–**G**). All data are presented as mean ± standard error of the mean. Ctrl = control; dMSN = direct pathway medium spiny neuron; iMSN = indirect pathway medium spiny neuron; Park = parkinsonian. See also Supplementary Fig. 5.

In people with PD, impulsivity induced by dopamine agonists such as PPX is usually reversible with dose reduction or discontinuation,^20^ suggesting that ICD may be mediated by drug-induced alterations in neural activity or connectivity. However, the specific brain areas and cell types dysregulated with agonist treatment are unclear. D2/3Rs are expressed across many brain regions including the striatum, amygdala and hippocampus.^21,48,49^ Specifically, the caudate nucleus (in primates) or dorsomedial striatum (DMS, in rodents) is a critical brain region for choice impulsivity.^16,50,51^ We next examined the responses of DMS neurons to PPX. D2Rs are densely expressed on indirect pathway neurons (iMSNs), and their activation is known to suppress activity in multiple brain regions.^23-25^ In previous work, we found that in DLS of hemiparkinsonian mice, dopamine agonists suppressed iMSN firing and (likely through synaptic mechanisms) increased dMSN firing.^21,25,52^ To determine how PPX affected dMSN and iMSN activity in the DMS, we performed optically identified single-unit electrophysiology in the DMS of both healthy and parkinsonian mice (Fig. 4A). To identify units as dMSNs or iMSNs, we expressed the excitatory opsin channelrhodopsin-2 (ChR2) selectively using D1-Cre or A2a-Cre mice, respectively^53,54^ and recorded responses to blue light pulses at the end of each session.^25,55^ First, we found that under baseline conditions, the dMSN and iMSN firing rates were comparable in control and parkinsonian mice (Fig. 4B and C). We next examined the effect of PPX (0.5 mg/kg), comparing baseline and post-injection periods. As delay discounting behaviour was tested between 4 and 5 h post-injection, we focused on the change between baseline and 4–5 h. In healthy mice, PPX immediately suppressed activity in both dMSNs and iMSNs, but firing returned to baseline levels by 4–5 h (Fig. 4D and E). We then classified individual optically identified units based on the difference between baseline and 4–5 h after PPX injection: ‘increase’, ‘decrease’ and ‘no change’ (no significant difference) types. In healthy mice, the majority of dMSNs and iMSNs showed no change, but a small proportion were inhibited or excited (Fig. 4D and E, insets). In parkinsonian mice, however, PPX caused bidirectional changes in optically identified MSN firing rates. PPX increased dMSN firing rates and decreased iMSN firing rates (Fig. 4F and G). Not only was this modulation more marked in parkinsonian mice (Fig. 4H–K), but the proportion of ‘increase’-type dMSNs and ‘decrease’-type iMSNs was increased (Fig. 4F and G, insets).

Importantly, similar patterns were seen in the larger unlabelled MSN pool (Supplementary Fig. 5A–D). Given the potential variability in firing rates over prolonged recordings, in separate experiments we injected sterile saline instead of PPX. Nearly all units showed no change in firing rate (Supplementary Fig. 5E and F). These findings demonstrate that MSNs are indeed bidirectionally dysregulated by PPX in parkinsonian mice, indicating aberrant MSN activity is a potential neural substrate for ICD.

### Chemogenetic manipulations of striatal neurons mimic the effect of pramipexole in parkinsonian mice

**Figure 5:**
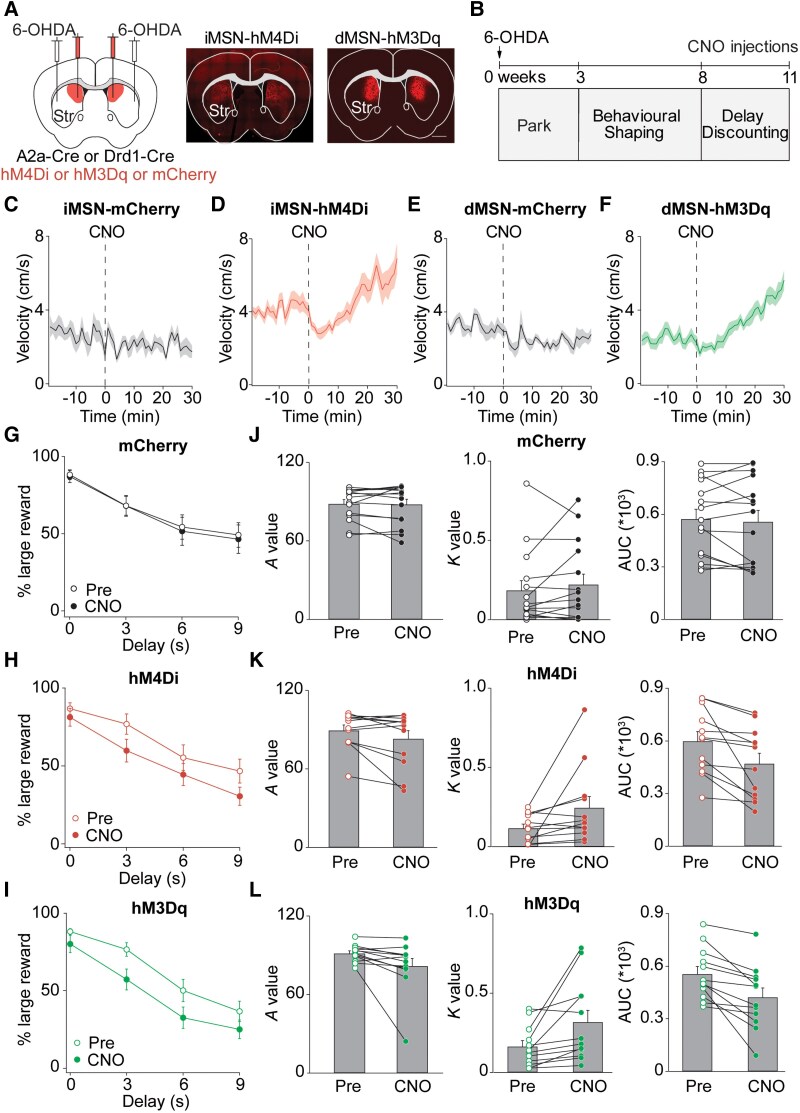
**Chemogenetic manipulations of striatal neurons mimic the effect of pramipexole in parkinsonian mice**. (**A**) *Left*: Injection schematic. *Right*: Post-mortem tissue showing mCherry expression in the dorsomedial striatum (DMS, red). (**B**) Experimental timeline. (**C**–**F**) Locomotor activity following intraperitoneal injection of CNO in A2a-Cre or D1-Cre parkinsonian mice expressing hM4Di, hM3Dq or mCherry (**C**, *n* = 8 animals; **D**, *n* = 13 animals; **E**, *n* = 5 animals; **F**, *n* = 12 animals). (**G**–**I**) Percentage delayed/large reward choices by either mCherry-, hM4Di- or hM3Dq-expressing mice at each delay during pre- (open circles) and post-CNO injection (filled circles) sessions (**G**, *n* = 15 animals, *P* > 0.99 at all delays; **H**, *n* = 12 animals, 0 s: *P* > 0.99, 3 s: *P* < 0.01, 6 s: *P* = 0.049, 9 s: *P* < 0.027; **I**, *n* = 12 animals, 0 s: *P* = 0.29, 3 s: *P* = 0.0002, 6 s: *P* = 0.0008, 9 s: *P* = 0.04. (**J**–**L**) *K*, *A* and AUC values from sessions before (pre-) and post-CNO administration in mice expressing mCherry, hM4Di or hM3Dq (mCherry: *n* = 15 animals, hM4Di: *n* = 12 animals, hM3Dq: *n* = 12 animals; mCherry, *A*: *P* = 0.89, *K*: *P* = 0.45, AUC: *P* = 0.56; hM4Di, *A*: *P* = 0.11, *K*: *P* = 0.02, AUC: *P* < 0.001; hM3Dq, *A*: *P* = 0.01, *K*: *P* < 0.01, AUC: *P* < 0.001. All data are presented as mean ± standard error of the mean. AUC = area under the curve; CNO = clozapine-N-oxide. See also Supplementary Fig. 6.

We found that PPX induced bidirectional changes in the firing rate of DMS striatal neurons, but these changes may or may not cause ICD-like changes in delay discounting behaviour. To test this hypothesis, we used a chemogenetic designer receptors exclusively activated by designer drugs (DREADD) approach. To mimic the effects of PPX on striatal activity, we used adeno-associated virus (AAV) to express the inhibitory DREADD hM4D_i_ (G_i_-coupled) in iMSNs, the excitatory DREADD hM3D_q_ (G_q_-coupled) in dMSNs, or a control fluorophore (mCherry) in dMSNs or iMSNs in the DMS of parkinsonian mice (Fig. 5A). To validate the use of hM4D_i_, we performed *ex vivo* whole-cell recordings from A2a-Cre;Drd2-GFP mice co-injected with Cre-dependent ChR2-eYFP and Cre-dependent hM4Di (Supplementary Fig. 6A), using the inhibitory connections between iMSNs and dMSNs as a functional readout of iMSN synaptic output. Brief light pulses evoked inhibitory postsynaptic currents (oIPSCs) in postsynaptic GFP-negative dMSNs (Supplementary Fig. 6A and B). Application of the DREADD agonist, clozapine-N-oxide (CNO), reduced oIPSC amplitude (Supplementary Fig. 6C), confirming that the G_i_-coupled DREADD inhibited iMSN output.^56^ To validate the use of hM3D_q_, we performed recordings from D1-Cre mice injected with Cre-dependent hM3D_q_ (Supplementary Fig. 6D). To measure dMSN excitability, we injected current steps of varying amplitude, and measured the output firing rate (Supplementary Fig. 6E). Application of CNO triggered a significant increase in firing rate (Supplementary Fig. 6F and G), confirming that the G_q_-coupled DREADD increased dMSN excitability.

We first tested whether chemogenetic inhibition of iMSNs or excitation of dMSNs, like PPX, could ameliorate parkinsonian locomotor deficits. In 6-OHDA-treated mice, injection of CNO increased locomotion in both hM4D_i_ and hM3D_q_ animals, but not in mCherry control animals, indicating a therapeutic effect (Fig. 5C–F). We then assessed whether chemogenetic inhibition of iMSNs or excitation of dMSNs was sufficient to cause impulsive decision-making. Parkinsonian hM4Di, hM3Dq or mCherry mice were assessed in the delay discounting task, before and after CNO treatments. Chemogenetic inhibition of iMSNs or excitation of dMSNs robustly shifted choices towards immediate/small rewards over delayed/large rewards in hM4D_i_-expressing or hM3D_q_-expressing but not mCherry control mice (Fig. 5G–I). Moreover, chemogenetic inhibition of indirect pathway or excitation of direct pathway significantly increased the *K* value and decreased AUC, consistent with a greater degree of impulsivity (Fig. 5J–L, middle and right). Interestingly, CNO did not impact the *A* value in the hM4D_i_-expressing group, but significantly reduced the *A* value in the hM3D_q_-expressing group, suggesting that manipulations of each pathway might differentially influence sensitivity to reward size (Fig. 5J–L, left). Together, these findings suggest that bidirectional chemogenetic modulation of striatal neurons within the DMS is sufficient to induce impulsive decision-making in parkinsonian mice in the absence of PPX treatment.

### Impulsivity develops with repeated pramipexole dosing, in parallel with changes in striatal activity

**Figure 6:**
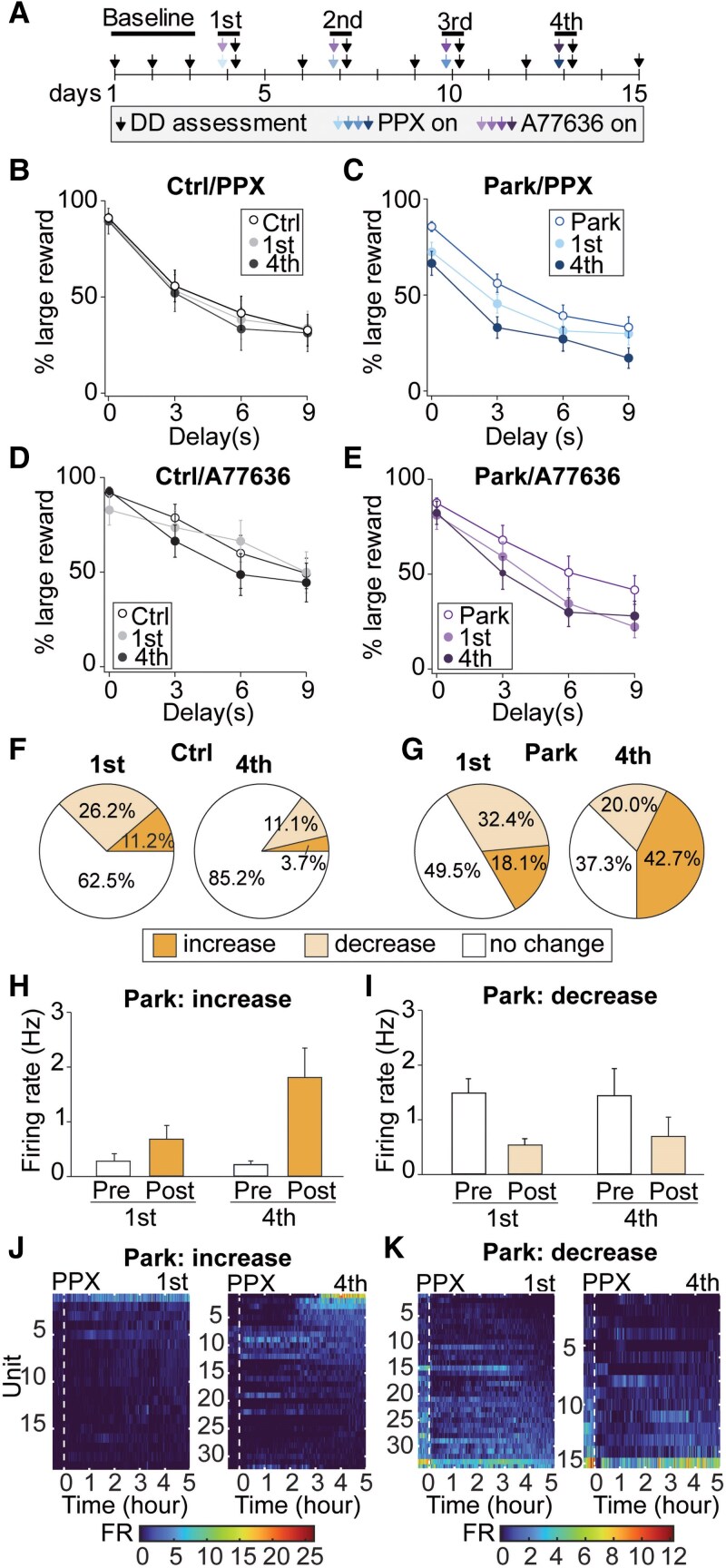
**Impulsivity develops with repeated pramipexole dosing, in parallel with changes in striatal activity.** (**A**–**E**) In healthy control (Ctrl) and parkinsonian (Park) mice, delay discounting behaviour was measured in PPX- or A77636-naive mice (baseline) and across 4 PPX or A77636 treatment sessions. (**A**) Experimental timeline. (**B**–**E**) Percentage of trials in which healthy control (**B** and **D**) and parkinsonian (**C** and **E**) mice chose the delayed/large reward across delays during baseline (open circles) and in the first and fourth PPX or A77636 session (filled circles) (**B**: *n* = 16 animals, **C**: *n* = 31 animals, **D**: *n* = 10 animals, **E**: *n* = 16 animals; **B**, first versus fourth: *P* > 0.99; **C**, first versus fourth: *P* < 0.0081; **D**, first versus fourth: *P* = 0.49; **E**, first versus fourth: *P* = 0.59; for other comparisons, refer to the Statistical Table). (**F** and **G**) Proportion of each response type during the first and fourth PPX session in control and parkinsonian mice [Ctrl: first (*n* = 7 animals, n = 80 cells) versus fourth (*n* = 3 animals, *n* = 27 cells), *P* = 0.11; Park: first (*n* = 10 animals, *n* = 105 cells) versus fourth (*n* = 8 animals, *n* = 75 cells), *P* = 0.002]. (**H** and **I**) Average firing rates before and after PPX among each response type in parkinsonian mice [**H**: first (*n* = 6 animals, *n* = 19 cells) versus fourth (*n* = 7 animals, *n* = 32 cells), *P* = 0.02; **I**: first (*n* = 9 animals, *n* = 34 cells) versus fourth (*n* = 5 animals, *n* = 15 cells), *P* = 0.89]. (**J** and **K**) Heat maps showing firing rates over time following PPX injection in parkinsonian mice. Responses during the first PPX session are on the left, the fourth session on the *right*, for neurons with an increase (**J**) or decrease (**K**) type response. Each row represents a single unit. All data are presented as mean ± standard error of the mean. PPX = pramipexole. See also Supplementary Figs 7 and 8.

Chronic dopamine replacement therapies (levodopa and dopamine agonists) may cause progressive involuntary movements and cognitive-behavioural dysfunction, highlighting plasticity at the behavioural level.^8,57-60^ At the cellular level, excitatory synaptic inputs onto MSNs are regulated by dopamine,^24,61-63^ and in parkinsonian mice, dopamine replacement therapies alter striatal synaptic plasticity.^64-66^ To determine whether chronic dopamine D1R or D2R agonist treatment caused progressive changes in delay discounting, we compared behaviour across four PPX or A77636 injection sessions (Fig. 6A). In healthy control mice, delay discounting remained consistent across sessions (Fig. 6B and D and Supplementary Fig. 7A and C). In parkinsonian mice, delay discounting changed over time: PPX injection induced a modest increase in impulsivity, which became more marked by the fourth session (Fig. 6C and Supplementary Fig. 7B). Conversely, the initial exposure to A77636 treatment resulted in increased impulsivity, which persisted across four consecutive treatments (Fig. 6D and Supplementary Fig. 7D).

Since PPX treatment resulted in progressive changes in delay discounting behaviour, we next examined whether alterations in MSN responses to PPX might underlie this behavioural plasticity. We compared how MSN firing changed in response to PPX between the first and fourth PPX session. In control mice, the proportion of response types was consistent across injection days (Fig. 6F and Supplementary Fig. 8A). However, in parkinsonian mice, the response types shifted over four sessions (Fig. 6G and Supplementary Fig. 8B). Notably, ‘increase’-type MSNs showed more dramatic increases in firing rate in response to the fourth (versus first) PPX injection (Fig. 6H and Supplementary Fig. 8C), while ‘decrease’-type MSNs responded similarly across sessions (Fig. 6I and Supplementary Fig. 8D). Chronic PPX treatment leads to a higher proportion of excited MSNs, each of which has a more dramatic response (Fig. 6J and Supplementary Fig. 8E). Conversely, the proportion of ‘decrease’-type MSNs falls over PPX treatment (Fig. 6K and Supplementary Fig. 8F). Together, these findings indicate that in parkinsonian animals, dopamine agonists lead to changes in striatal firing which contribute to the development of ICD-like behaviour.

## Discussion

Here, we established a mouse model of ICD in PD and investigated the role of aberrant striatal activity in impulsive decision-making. We found that either PPX, a widely used D2/3R agonist, or A77636, a D1R agonist, induced impulsive decision-making in parkinsonian mice, as assessed by a delay discounting task. Dopaminergic denervation in the associative (cognitive) subregion of the striatum was positively correlated with greater delay discounting behaviour, suggesting its critical role. PPX caused bidirectional changes in iMSN/dMSN firing rates in the DMS of parkinsonian mice, while having minimal effects in healthy mice. Chronic PPX potentiated changes in striatal physiology and decision-making behaviour. Furthermore, excitation of direct pathway or inhibition of indirect pathway output was sufficient to cause impulsive decision-making, mimicking the effects of PPX. Our study is the first to perform physiological recordings in a clinically relevant mouse model of ICD, and to link a specific striatal circuity to ICD.

We found that in mildly parkinsonian mice, dopamine agonist treatment reproduced key clinical features of ICD, such as the increased risk in PD patients (versus other individuals) and the medication dependence of impulsive behaviour.^10-12^ However, significant motor deficits can create confounds in operant tasks, preventing accurate assessment of cognitive-behavioural phenotypes in mouse models of PD. To overcome this potential obstacle, we adapted a mouse model of PD with relatively restricted bilateral dopamine depletion, reminiscent of what is seen in early PD, when dopamine agonists are most likely to be employed. While this model showed mild motor deficits, animals could still perform our task, and motivational metrics were comparable to those in control mice. Another key variable in our model was the dose of dopamine agonist. Prior work indicates higher doses of D2/3R agonists have reinforcing properties even in intact animals.^67,68^ We calibrated the agonist to provide motor benefit while avoiding supratherapeutic dosing. Although D2/3R agonists are considered a strong risk factor for ICD, other dopamine replacement therapies, such as levodopa, induce ICD (albeit at lower rates), suggesting the involvement of both D1R and D2R signalling.^69^ As with the D2R agonist, we chose a low dose of a D1R agonist to produce therapeutic locomotor effects in parkinsonian mice, as higher doses of D1R agonists induced aversive behaviour in 6-OHDA-lesioned rats.^68^ We found that the D1R agonist A77636 produced impulsivity only in parkinsonian mice, consistent with our findings with PPX. Overall, we believe that the risk of ICD is associated with abnormal dopamine signalling in a vulnerable neural substrate, which would explain the differences between healthy and parkinsonian mice in their behavioural and physiological responses to PPX.

In addition to the type and dose of medication to treat motor symptoms in PD, other risk factors for ICD include sex and the drug formulation/route.^5,41^ We found impulsivity developed in both male and female mice after PPX treatment, to a similar degree across groups. These results mirror the observation that in people with PD, ICD frequency is similar between men and women. Interestingly, however, specific types of ICD show clear sex differences.^41,70^ For example, men are more likely than women to develop compulsive sexual behaviour, but less likely to engage in compulsive shopping.^71^ The behavioural assay used here taps into fundamental decision-making processes, which may not differ markedly between males and females. Previous clinical evidence suggests that long-acting treatments, such as transdermal patch or pump formulations, may lower ICD risk compared with pulsatile receptor stimulation.^72,73^ These treatments may achieve lower peak doses or drive slower changes in dopamine signalling, reducing the risk of ICD. When comparing short- and long-acting dopamine replacement therapies, it is important to note that, in clinical practice, long-acting drugs are likely to achieve lower peak plasma levels than short-acting ones, albeit for longer periods. Given that ICD has been shown to be dose dependent, with lower doses associated with reduced risk, this may explain the observed differences between formulations.^20,74^ To better understand the impact of pharmacokinetic properties of dopamine agonists on ICD, future studies could use subcutaneous pumps to continuously deliver PPX in animal models.

Altered delay discounting behaviour can be driven by changes in how reward magnitude, time, and/or reward/delay trade-offs are processed.^75^ As in previous studies, we used Herrnstein's hyperbolic model V = *A*/(1 + *K*D) to fit behaviour.^32,36,37^ In this equation, ‘D’ represents the delay, and ‘*A*’ and ‘*K*’ factors reflect sensitivity to reward magnitude and delay durations, respectively. We found that PPX increased the *K* value in parkinsonian mice, as has been seen in PD patients with ICD.^6,11,12^ These findings suggest PPX-treated mice are more intolerant of waiting, even for a larger reward. Impulsivity also correlates with poor temporal discrimination in rats and humans.^75,76^ Interestingly, in a subset of parkinsonian mice, PPX also decreased the *A* value, suggesting impaired processing of reward magnitude. While the small number of delay discounting studies in PD/ICD have not shown changes in the *A* value, heightened sensitivity to reward magnitude has been implicated in driving ICD symptoms in certain susceptible individuals.^9^ Moreover, other studies of impulsivity suggest reward magnitude discrimination is crucial in driving impulsive choice.^39^ However, one limitation is that *A* and *K* values may not be fully independent, particularly in parkinsonian mice, as reward magnitude and temporal sensitivity can interact. For instance, mice with deficits in reward magnitude sensitivity may also have impaired temporal perception, as both are linked to the encoding of reward outcomes. To account for this limitation, we additionally computed the AUC as a global measure of discounting.

While deficits in reward size or temporal sensitivity (changes in *A* and *K* values) are key factors contributing to impulsive decision-making, other factors, such as reduced motivation, have also been implicated in impulsivity across neuropsychiatric conditions.^77,78^ However, reduced motivation or anhedonia are unlikely to explain the behavioural changes we observed. First, omission rates were similarly low in both PPX-naive and PPX-treated mice, indicating high levels of task engagement. Anhedonia would be expected to drive greater omissions, as has been seen in other models and tasks.^79^ Second, parkinsonian mice treated with PPX exhibited shorter latencies to trigger immediate small rewards. This suggests vigorous seeking of immediate small rewards, rather than a general motivational deficit, which would instead be expected to slow responses. Third, previous studies show that PPX can enhance, rather than reduce, motivational vigour in human studies,^80^ and improve motivational symptoms in PD patients.^81^

PPX has multiple sites of action, and is known to reduce dopamine release via activation of D2 autoreceptors,^82-84^ which in turn can reduce locomotor behaviour in a dose-dependent fashion in healthy rodents.^31,85^ This effect is likely attenuated in parkinsonian mice, where only ∼30%–50% of dopaminergic terminals remain, but is probably responsible for the decrease in locomotor activity. In more progressive PD, the number of remaining dopamine terminals available for presynaptic autoregulation is likely even lower. Indeed, in a more severe unilateral 6-OHDA mouse model of PD, dopamine agonists induce almost instantaneous increases in locomotion, dMSN firing and parallel decreases in iMSN firing, consistent with an absence of this presynaptic autoreceptor effect.^25^ In our model, we selected an intermediate dose of PPX that did not induce sustained freezing behaviour, and which at later time points improved motor deficits on the rotarod test. We targeted our cognitive assays for this later, therapeutic period, when we suspect the impact of PPX on presynaptic dopamine release is minimal, particularly in partially dopamine-depleted mice. For these reasons, we do not believe that the presynaptic D2R signalling on dopamine terminals is a major driver of impulsivity in the model.

We found that in PPX-treated parkinsonian mice, greater dopaminergic denervation in the dorsomedial striatum (DMS), but not dorsolateral striatum (DLS), was associated with changes in reward magnitude processing (*A* value). These findings are in line with studies that indicate the DMS encodes reward magnitudes.^86^ Furthermore, they support the hypothesis that ICD arises from the action of dopamine replacement therapy on vulnerable circuitry, with disease severity differences influencing susceptibility.^9^ A more comprehensive rodent study, examining varying degrees of dopamine depletion across different striatal subregions and assessing behavioural impulsivity, will be necessary to test this hypothesis.

Consistent with the effects of levodopa on striatal activity in parkinsonian mice,^25^ we found that PPX induced bidirectional changes in striatal activity. This observation suggests that imbalanced striatal activity is a key driver of ICD. However, the risk of ICD is lower with levodopa,^41^ likely due to differences in receptor activation profiles: levodopa leads to dopamine release, which in turn would tend to activate both D1Rs and D2Rs, whereas dopamine D2R agonists like PPX selectively target D2Rs (and D3Rs). Other mechanisms may also contribute to the difference, including the effects of dopamine D2R agonists outside iMSNs and/or the striatum. For example, PPX may act on D2Rs expressed in frontal cortical neurons and their terminals in the striatum, which are critical for decision-making.^52,87,88^ Further studies to determine molecular adaptations of MSNs triggered by dopamine depletion and PPX treatment could reveal additional mechanisms in ICD.

Interestingly, we found that mild dopamine depletion alone did not markedly change overall MSN firing rates, suggesting homeostatic adaptations, such as diminished dopamine reuptake capacity within partially denervated striatum,^89^ may help maintain baseline activity within normal limits. Homeostatic adaptations may also account for the similar baseline delay discounting observed in parkinsonian and healthy mice. However, PPX tipped this fragile balance, resulting in ICD-like behaviour. We hypothesize ICD arises in part from the interaction of PPX with the chronically dopamine-depleted striatum. Previous work has identified many alterations to striatal signalling molecules and physiological properties in people with PD and animal models of PD.^90,91^ These alterations include upregulation of D2Rs,^92,93^ which may explain the more pronounced suppression of iMSN firing in parkinsonian mice following PPX treatment. The sensitivity of the dMSN to PPX could be mediated by suppression of collateral inhibition from iMSNs.^94,95^ We also found that over multiple doses, physiological responses to PPX potentiated. This phenomenon may relate to additional adaptations in striatal circuitry, including alterations in synaptic plasticity, that have been seen with repeated dopaminergic treatments in animal models of psychostimulant sensitization, chronic PPX treatment^96-98^ or levodopa-induced dyskinesia.^66^

We found that in parkinsonian mice, bidirectional chemogenetic manipulations of DMS dMSNs or iMSNs induced an ICD-like phenotype, with more pronounced delay discounting. This observation is in line with evidence that the associative striatum (caudate nucleus in primates, or DMS in rodents) plays a significant role in mediating impulsive decision-making. Pharmacological and electrophysiological studies have linked this region to delay discounting and decision-making in healthy animals.^13,16,99,100^ It is also consistent with the pharmacology of dopaminergic agonists (both D1R and D2R agonists) on striatal circuitry. Dopaminergic agonists would be predicted to reduce indirect pathway output, while activating direct pathway neurons, in part via local inhibitory collaterals.^56^ This prediction is supported by prior work demonstrating D2/3R agonists increase activity in the globus pallidus, and decrease activity in the substantia nigra reticulata in monkeys.^101,102^ Chemogenetic inhibition of iMSNs or excitation of dMSNs may mimic key effects of dopamine agonists, leading to impulsive decision-making. However, dopamine agonists and chemogenetic manipulation of striatal circuitry had some shared and some distinct behavioural effects. PPX and chemogenetic excitation of dMSNs increased *K*-values and decreased *A*-values, whereas chemogenetic inhibition of iMSNs increased *K*-values without significantly altering *A*-values. This discrepancy may be due to the fact that PPX acts on both D2R and D3R. D3R co-localize with D1Rs in the ventral striatum,^103^ but show ectopic expression in the dorsal striatum of parkinsonian animals treated without dopamine replacement therapy.^66^

Together, our results suggest a key potential mechanism for impulsive decision-making in ICD: dysregulated dMSN and iMSN activity in parkinsonian animals treated with dopamine agonist medication. This insight could inform the use of dopamine replacement therapy with a goal of preventing or ameliorating ICD.

## Supplementary Material

awaf312_Supplementary_Data

## Data Availability

Data and original code have been deposited at Zenodo and are publicly available as of the date of publication (https://doi.org/10.5281/zenodo.15258978). Raw *in vivo* electrophysiology datasets have been deposited in DANDI (https://dandiarchive.org/dandiset/001177) and are publicly available as of publication. Detailed protocols and analysis code are listed within the ‘Materials and methods’ section.

## References

[1] El Massioui N, Lamirault C, Yague S, et al Impaired decision making and loss of inhibitory-control in a rat model of Huntington disease. Front Behav Neurosci. 2016;10:204. 27833538 10.3389/fnbeh.2016.00204 PMC5080295

[2] Winstanley CA, Eagle DM, Robbins TW. Behavioral models of impulsivity in relation to ADHD: Translation between clinical and preclinical studies. Clin Psychol Rev. 2006;26:379–395. 16504359 10.1016/j.cpr.2006.01.001 PMC1892795

[3] Jankovic J . Parkinson’s disease: Clinical features and diagnosis. J Neurol Neurosurg Psychiatry. 2008;79:368–376. 18344392 10.1136/jnnp.2007.131045

[4] Augustine A, Winstanley CA, Krishnan V. Impulse control disorders in Parkinson’s disease: From bench to bedside. Front Neurosci. 2021;15:654238. 33790738 10.3389/fnins.2021.654238 PMC8006437

[5] Weintraub D, Mamikonyan E. Impulse control disorders in Parkinson’s disease. Am J Psychiatry. 2019;176:5–11. 30848949 10.1176/appi.ajp.2018.18040465

[6] Voon V, Reynolds B, Brezing C, et al Impulsive choice and response in dopamine agonist-related impulse control behaviors. Psychopharmacology (Berl). 2010;207:645–659. 19838863 10.1007/s00213-009-1697-y PMC3676926

[7] Leeman RF, Potenza MN. Impulse control disorders in Parkinson’s disease: Clinical characteristics and implications. Neuropsychiatry (London). 2011;1:133–147. 21709778 10.2217/npy.11.11 PMC3120055

[8] Jimenez-Urbieta H, Gago B, Quiroga-Varela A, et al Pramipexole-induced impulsivity in mildparkinsonian rats: A model of impulse control disorders in Parkinson’s disease. Neurobiol Aging. 2019;75:126–135. 30572183 10.1016/j.neurobiolaging.2018.11.021

[9] Drew DS, Muhammed K, Baig F, et al Dopamine and reward hypersensitivity in Parkinson’s disease with impulse control disorder. Brain. 2020;143:2502–2518. 32761061 10.1093/brain/awaa198 PMC7447523

[10] Pennisi P, Salehinejad MA, Corso AM, Merlo EM, Avenanti A, Vicario CM. Delay discounting in Parkinson’s disease: A systematic review and meta-analysis. Behav Brain Res. 2023;436:114101. 36087861 10.1016/j.bbr.2022.114101

[11] Housden CR, O’Sullivan SS, Joyce EM, Lees AJ, Roiser JP. Intact reward learning but elevated delay discounting in Parkinson’s disease patients with impulsive-compulsive spectrum behaviors. Neuropsychopharmacology. 2010;35:2155–2164. 20631686 10.1038/npp.2010.84 PMC3055316

[12] Izzo VA, Donati MA, Torre E, Ramat S, Primi C. Impulse control disorders in Parkinson’s disease versus in healthy controls: A different predictive model. J Neuropsychol. 2020;14:318–332. 31423741 10.1111/jnp.12193

[13] Cai X, Kim S, Lee D. Heterogeneous coding of temporally discounted values in the dorsal and ventral striatum during intertemporal choice. Neuron. 2011;69:170–182. 21220107 10.1016/j.neuron.2010.11.041 PMC3034314

[14] Joutsa J, Voon V, Johansson J, Niemela S, Bergman J, Kaasinen V. Dopaminergic function and intertemporal choice. Transl Psychiatry. 2015;5:e520–e520. 25734513 10.1038/tp.2015.12 PMC4354344

[15] Wenzel JM, Zlebnik NE, Patton MH, et al Selective chemogenetic inactivation of corticoaccumbal projections disrupts trait choice impulsivity. Neuropsychopharmacology. 2023;48:1821–1831. 37208501 10.1038/s41386-023-01604-5 PMC10579332

[16] Martinez E, Pasquereau B, Saga Y, Metereau E, Tremblay L. The anterior caudate nucleus supports impulsive choices triggered by pramipexole. Mov Disord. 2020;35:296–305. 31737954 10.1002/mds.27898

[17] Webber ES, Mankin DE, Cromwell HC. Striatal activity and reward relativity: Neural signals encoding dynamic outcome valuation. eNeuro. 2016;3:ENEURO.0022-16.2016. 10.1523/ENEURO.0022-16.2016 PMC5089537 27822506

[18] Shin JH, Kim D, Jung MW. Differential coding of reward and movement information in the dorsomedial striatal direct and indirect pathways. Nat Commun. 2018;9:404. 29374173 10.1038/s41467-017-02817-1 PMC5786099

[19] Toso A, Reinartz S, Pulecchi F, Diamond ME. Time coding in rat dorsolateral striatum. Neuron. 2021;109:3663–3673.e6. 34508666 10.1016/j.neuron.2021.08.020

[20] Kelly MJ, Baig F, Hu MTM, Okai D. Spectrum of impulse control behaviours in Parkinson’s disease: Pathophysiology and management. J Neurol Neurosurg Psychiatry. 2020;91:703–711. 32354771 10.1136/jnnp-2019-322453

[21] Gerfen CR, Engber TM, Mahan LC, et al D1 and D2 dopamine receptor-regulated gene expression of striatonigral and striatopallidal neurons. Science. 1990;250:1429–1432. 2147780 10.1126/science.2147780

[22] Albin RL, Young AB, Penney JB. The functional anatomy of basal ganglia disorders. Trends Neurosci. 1989;12:366–375. 2479133 10.1016/0166-2236(89)90074-x

[23] DeLong MR . Primate models of movement disorders of basal ganglia origin. Trends Neurosci. 1990;13:281–285. 1695404 10.1016/0166-2236(90)90110-v

[24] Planert H, Berger TK, Silberberg G. Membrane properties of striatal direct and indirect pathway neurons in mouse and rat slices and their modulation by dopamine. PLoS One. 2013;8:e57054. 23469183 10.1371/journal.pone.0057054 PMC3585935

[25] Ryan MB, Bair-Marshall C, Nelson AB. Aberrant striatal activity in Parkinsonism and levodopa-induced dyskinesia. Cell Rep. 2018;23:3438–3446.e5. 29924988 10.1016/j.celrep.2018.05.059 PMC6407866

[26] Maltese M, March JR, Bashaw AG, Tritsch NX. Dopamine differentially modulates the size of projection neuron ensembles in the intact and dopamine-depleted striatum. Elife. 2021;10:e68041. 33983121 10.7554/eLife.68041 PMC8163504

[27] Parker JG, Marshall JD, Ahanonu B, et al Diametric neural ensemble dynamics in parkinsonian and dyskinetic states. Nature. 2018;557:177–182. 29720658 10.1038/s41586-018-0090-6 PMC6526726

[28] Liang L, DeLong MR, Papa SM. Inversion of dopamine responses in striatal medium spiny neurons and involuntary movements. J Neurosci. 2008;28:7537–7547. 18650331 10.1523/JNEUROSCI.1176-08.2008 PMC3343722

[29] Alcacer C, Andreoli L, Sebastianutto I, Jakobsson J, Fieblinger T, Cenci MA. Chemogenetic stimulation of striatal projection neurons modulates responses to Parkinson’s disease therapy. J Clin Invest. 2017;127:720–734. 28112685 10.1172/JCI90132 PMC5272195

[30] Stowe RL, Ives NJ, Clarke C, et al Dopamine agonist therapy in early Parkinson’s disease. Cochrane Database Syst Rev. 2008;2:CD006564. 10.1002/14651858.CD006564.pub2 PMC12740267 18425954

[31] Chang WL, Geyer MA, Buell MR, Weber M, Swerdlow NR. The effects of pramipexole on prepulse inhibition and locomotor activity in C57BL/6J mice. Behav Pharmacol. 2010;21:135–143. 20215963 10.1097/FBP.0b013e328337be7e PMC2890261

[32] Chung SH, Herrnstein RJ. Choice and delay of reinforcement. J Exp Anal Behav. 1967;10:67–74. 16811307 10.1901/jeab.1967.10-67 PMC1338319

[33] Ondo WG, Lai D. Predictors of impulsivity and reward seeking behavior with dopamine agonists. Parkinsonism Relat Disord. 2008;14:28–32. 17702628 10.1016/j.parkreldis.2007.05.006

[34] Mohebi A, Pettibone JR, Hamid AA, et al Dissociable dopamine dynamics for learning and motivation. Nature. 2019;570:65–70. 31118513 10.1038/s41586-019-1235-y PMC6555489

[35] Rowe JB, Eckstein D, Braver T, Owen AM. How does reward expectation influence cognition in the human brain? J Cogn Neurosci. 2008;20:1980–1992. 18416677 10.1162/jocn.2008.20140 PMC3886193

[36] Berns GS, Laibson D, Loewenstein G. Intertemporal choice–toward an integrative framework. Trends Cogn Sci. 2007;11:482–488. 17980645 10.1016/j.tics.2007.08.011

[37] Herrnstein RJ . Relative and absolute strength of response as a function of frequency of reinforcement. J Exp Anal Behav. 1961;4:267–272. 13713775 10.1901/jeab.1961.4-267 PMC1404074

[38] Madden GJ, Ewan EE, Lagorio CH. Toward an animal model of gambling: Delay discounting and the allure of unpredictable outcomes. J Gambl Stud. 2007;23:63–83. 17171542 10.1007/s10899-006-9041-5

[39] Marshall AT, Kirkpatrick K. Mechanisms of impulsive choice: III. The role of reward processes. Behav Processes. 2016;123:134–148. 26506254 10.1016/j.beproc.2015.10.013 PMC4729665

[40] Myerson J, Green L, Warusawitharana M. Area under the curve as a measure of discounting. J Exp Anal Behav. 2001;76:235–243. 11599641 10.1901/jeab.2001.76-235 PMC1284836

[41] Weintraub D, Koester J, Potenza MN, et al Impulse control disorders in Parkinson disease: A cross-sectional study of 3090 patients. Arch Neurol. 2010;67:589–595. 20457959 10.1001/archneurol.2010.65

[42] Kebabian JW, Britton DR, DeNinno MP, et al A-77636: A potent and selective dopamine D1 receptor agonist with antiparkinsonian activity in marmosets. Eur J Pharmacol. 1992;229:203–209. 1362704 10.1016/0014-2999(92)90556-j

[43] Self DW, Belluzzi JD, Kossuth S, Stein L. Self-administration of the D1 agonist SKF 82958 is mediated by D1, not D2, receptors. Psychopharmacology (Berl). 1996;123:303–306. 8867867 10.1007/BF02246638

[44] Grech DM, Spealman RD, Bergman J. Self-administration of D1 receptor agonists by squirrel monkeys. Psychopharmacology (Berl). 1996;125:97–104. 8783382 10.1007/BF02249407

[45] Ansari MF, Prasad S, Bhardwaj S, et al Morphometric alterations of the mesocorticolimbic network in Parkinson’s disease with impulse control disorders. J Neural Transm (Vienna). 2024;131:229–237. 38216706 10.1007/s00702-023-02735-1

[46] Ruitenberg MFL, Wu T, Averbeck BB, Chou KL, Koppelmans V, Seidler RD. Impulsivity in Parkinson’s disease is associated with alterations in affective and sensorimotor striatal networks. Front Neurol. 2018;9:279. 29755401 10.3389/fneur.2018.00279 PMC5932175

[47] Carriere N, Lopes R, Defebvre L, Delmaire C, Dujardin K. Impaired corticostriatal connectivity in impulse control disorders in Parkinson disease. Neurology. 2015;84:2116–2123. 25925985 10.1212/WNL.0000000000001619

[48] Kim B, Yoon S, Nakajima R, et al Dopamine D2 receptor-mediated circuit from the central amygdala to the bed nucleus of the stria terminalis regulates impulsive behavior. Proc Natl Acad Sci U S A. 2018;115:E10730–E10739. 30348762 10.1073/pnas.1811664115 PMC6233075

[49] Dubovyk V, Manahan-Vaughan D. Gradient of expression of dopamine D2 receptors along the dorso-ventral axis of the hippocampus. Front Synaptic Neurosci. 2019;11:28. 31680927 10.3389/fnsyn.2019.00028 PMC6803426

[50] Kim B, Im HI. The role of the dorsal striatum in choice impulsivity. Ann N Y Acad Sci. 2019;1451:92–111. 30277562 10.1111/nyas.13961

[51] Gu L, Shu H, Wang Y, Xu H. Exploring brain changes of impulse control disorders in Parkinson’s disease: An ALE study. Front Aging Neurosci. 2022;14:966525. 36110428 10.3389/fnagi.2022.966525 PMC9468821

[52] Wang H, Pickel VM. Dopamine D2 receptors are present in prefrontal cortical afferents and their targets in patches of the rat caudate-putamen nucleus. J Comp Neurol. 2002;442:392–404. 11793342 10.1002/cne.10086

[53] Gerfen CR, Paletzki R, Heintz N. GENSAT BAC cre-recombinase driver lines to study the functional organization of cerebral cortical and basal ganglia circuits. Neuron. 2013;80:1368–1383. 24360541 10.1016/j.neuron.2013.10.016 PMC3872013

[54] Gong S, Doughty M, Harbaugh CR, et al Targeting Cre recombinase to specific neuron populations with bacterial artificial chromosome constructs. J Neurosci. 2007;27:9817–9823. 17855595 10.1523/JNEUROSCI.2707-07.2007 PMC6672645

[55] Kravitz AV, Owen SF, Kreitzer AC. Optogenetic identification of striatal projection neuron subtypes during in vivo recordings. Brain Res. 2013;1511:21–32. 23178332 10.1016/j.brainres.2012.11.018 PMC3594574

[56] Dobbs LK, Kaplan AR, Lemos JC, Matsui A, Rubinstein M, Alvarez VA. Dopamine regulation of lateral inhibition between striatal neurons gates the stimulant actions of cocaine. Neuron. 2016;90:1100–1113. 27181061 10.1016/j.neuron.2016.04.031 PMC4891261

[57] Ahlskog JE, Muenter MD. Frequency of levodopa-related dyskinesias and motor fluctuations as estimated from the cumulative literature. Mov Disord. 2001;16:448–458. 11391738 10.1002/mds.1090

[58] Bodi N, Keri S, Nagy H, et al Reward-learning and the novelty-seeking personality: A between- and within-subjects study of the effects of dopamine agonists on young Parkinson’s patients. Brain. 2009;132(Pt 9):2385–2395. 19416950 10.1093/brain/awp094 PMC2766178

[59] Rokosik SL, Napier TC. Pramipexole-induced increased probabilistic discounting: Comparison between a rodent model of Parkinson’s disease and controls. Neuropsychopharmacology. 2012;37:1397–1408. 22257895 10.1038/npp.2011.325 PMC3327845

[60] Abler B, Hahlbrock R, Unrath A, Gron G, Kassubek J. At-risk for pathological gambling: Imaging neural reward processing under chronic dopamine agonists. Brain. 2009;132:2396–2402. 19567700 10.1093/brain/awp170

[61] Gerfen CR, Surmeier DJ. Modulation of striatal projection systems by dopamine. Annu Rev Neurosci. 2011;34:441–466. 21469956 10.1146/annurev-neuro-061010-113641 PMC3487690

[62] Fieblinger T, Graves SM, Sebel LE, et al Cell type-specific plasticity of striatal projection neurons in parkinsonism and L-DOPA-induced dyskinesia. Nat Commun. 2014;5:5316. 25360704 10.1038/ncomms6316 PMC4431763

[63] Lahiri AK, Bevan MD. Dopaminergic transmission rapidly and persistently enhances excitability of D1 receptor-expressing striatal projection neurons. Neuron. 2020;106:277–290.e6. 32075716 10.1016/j.neuron.2020.01.028 PMC7182485

[64] Shen W, Flajolet M, Greengard P, Surmeier DJ. Dichotomous dopaminergic control of striatal synaptic plasticity. Science. 2008;321:848–851. 18687967 10.1126/science.1160575 PMC2833421

[65] Calabresi P, Ghiglieri V, Mazzocchetti P, Corbelli I, Picconi B. Levodopa-induced plasticity: A double-edged sword in Parkinson’s disease? Philos Trans R Soc Lond B Biol Sci. 2015;370:20140184. 26009763 10.1098/rstb.2014.0184 PMC4455753

[66] Bordet R, Ridray S, Carboni S, Diaz J, Sokoloff P, Schwartz JC. Induction of dopamine D3 receptor expression as a mechanism of behavioral sensitization to levodopa. Proc Natl Acad Sci U S A. 1997;94:3363–3367. 9096399 10.1073/pnas.94.7.3363 PMC20375

[67] Engeln M, Ahmed SH, Vouillac C, Tison F, Bezard E, Fernagut PO. Reinforcing properties of pramipexole in normal and parkinsonian rats. Neurobiol Dis. 2013;49:79–86. 22940424 10.1016/j.nbd.2012.08.005

[68] Zengin-Toktas Y, Authier N, Denizot H, et al Motivational properties of D2 and D3 dopamine receptors agonists and cocaine, but not with D1 dopamine receptors agonist and L-dopa, in bilateral 6-OHDA-lesioned rat. Neuropharmacology. 2013;70:74–82. 23347953 10.1016/j.neuropharm.2012.12.011

[69] O’Sullivan SS, Evans AH, Lees AJ. Punding in Parkinson’s disease. Pract Neurol. 2007;7:397–399. 18024780 10.1136/jnnp.2007.129015

[70] Koran LM, Faber RJ, Aboujaoude E, Large MD, Serpe RT. Estimated prevalence of compulsive buying behavior in the United States. Am J Psychiatry. 2006;163:1806–1812. 17012693 10.1176/ajp.2006.163.10.1806

[71] Kuzma JM, Black DW. Epidemiology, prevalence, and natural history of compulsive sexual behavior. Psychiatr Clin North Am. 2008;31:603–611. 18996301 10.1016/j.psc.2008.06.005

[72] Rizos A, Sauerbier A, Antonini A, et al A European multicentre survey of impulse control behaviours in Parkinson’s disease patients treated with short- and long-acting dopamine agonists. Eur J Neurol. 2016;23:1255–1261. 27170229 10.1111/ene.13034

[73] Barbosa P, Lees AJ, Magee C, Djamshidian A, Warner TT. A retrospective evaluation of the frequency of impulsive compulsive behaviors in Parkinson’s disease patients treated with continuous waking day apomorphine pumps. Mov Disord Clin Pract. 2017;4:323–328. 30363495 10.1002/mdc3.12416 PMC6174511

[74] Gallagher DA, O’Sullivan SS, Evans AH, Lees AJ, Schrag A. Pathological gambling in Parkinson’s disease: Risk factors and differences from dopamine dysregulation. An analysis of published case series. Mov Disord. 2007;22:1757–1763. 17580327 10.1002/mds.21611

[75] Galtress T, Garcia A, Kirkpatrick K. Individual differences in impulsive choice and timing in rats. J Exp Anal Behav. 2012;98:65–87. 22851792 10.1901/jeab.2012.98-65 PMC3408726

[76] Baumann AA, Odum AL. Impulsivity, risk taking, and timing. Behav Processes. 2012;90:408–414. 22542458 10.1016/j.beproc.2012.04.005 PMC3897391

[77] Volkow ND, Wang GJ, Newcorn JH, et al Motivation deficit in ADHD is associated with dysfunction of the dopamine reward pathway. Mol Psychiatry. 2011;16:1147–1154. 20856250 10.1038/mp.2010.97 PMC3010326

[78] File D, Bothe B, File B, Demetrovics Z. The role of impulsivity and reward deficiency in “liking” and “wanting” of potentially problematic behaviors and substance uses. Front Psychiatry. 2022;13:820836. 35546934 10.3389/fpsyt.2022.820836 PMC9083266

[79] Vouillac-Mendoza C, Durand A, Ahmed SH, Guillem K. Knowledge by omission: The significance of omissions in the 5-choice serial reaction time task. Psychopharmacology (Berl). 2024;241:1319–1328. 38443605 10.1007/s00213-024-06564-2

[80] Au-Yeung SK, Halahakoon DC, Kaltenboeck A, Cowen P, Browning M, Manohar SG. The effects of pramipexole on motivational vigour during a saccade task: A placebo-controlled study in healthy adults. Psychopharmacology (Berl). 2024;241:1365–1375. 38494550 10.1007/s00213-024-06567-z PMC11199222

[81] Leentjens AF, Koester J, Fruh B, Shephard DT, Barone P, Houben JJ. The effect of pramipexole on mood and motivational symptoms in Parkinson’s disease: A meta-analysis of placebo-controlled studies. Clin Ther. 2009;31:89–98. 19243709 10.1016/j.clinthera.2009.01.012

[82] Carter AJ, Muller RE. Pramipexole, a dopamine D2 autoreceptor agonist, decreases the extracellular concentration of dopamine in vivo. Eur J Pharmacol. 1991;200:65–72. 1685123 10.1016/0014-2999(91)90666-e

[83] Usiello A, Baik JH, Rouge-Pont F, et al Distinct functions of the two isoforms of dopamine D2 receptors. Nature. 2000;408:199–203. 11089973 10.1038/35041572

[84] De Mei C, Ramos M, Iitaka C, Borrelli E. Getting specialized: Presynaptic and postsynaptic dopamine D2 receptors. Curr Opin Pharmacol. 2009;9:53–58. 19138563 10.1016/j.coph.2008.12.002 PMC2710814

[85] Chang WL, Breier MR, Yang A, Swerdlow NR. Disparate effects of pramipexole on locomotor activity and sensorimotor gating in Sprague-Dawley rats. Pharmacol Biochem Behav. 2011;99:634–638. 21683731 10.1016/j.pbb.2011.06.002 PMC5946323

[86] Delgado MR, Locke HM, Stenger VA, Fiez JA. Dorsal striatum responses to reward and punishment: Effects of valence and magnitude manipulations. Cogn Affect Behav Neurosci. 2003;3:27–38. 12822596 10.3758/cabn.3.1.27

[87] Wang W, Dever D, Lowe J, et al Regulation of prefrontal excitatory neurotransmission by dopamine in the nucleus accumbens core. J Physiol. 2012;590:3743–3769. 22586226 10.1113/jphysiol.2012.235200 PMC3476631

[88] Dalley JW, Everitt BJ, Robbins TW. Impulsivity, compulsivity, and top-down cognitive control. Neuron. 2011;69:680–694. 21338879 10.1016/j.neuron.2011.01.020

[89] Garris PA, Walker QD, Wightman RM. Dopamine release and uptake rates both decrease in the partially denervated striatum in proportion to the loss of dopamine terminals. Brain Res. 1997;753:225–234. 9125407 10.1016/s0006-8993(97)00003-6

[90] Corvol JC, Muriel MP, Valjent E, et al Persistent increase in olfactory type G-protein alpha subunit levels may underlie D1 receptor functional hypersensitivity in Parkinson disease. J Neurosci. 2004;24:7007–7014. 15295036 10.1523/JNEUROSCI.0676-04.2004 PMC6729591

[91] Guigoni C, Doudnikoff E, Li Q, Bloch B, Bezard E. Altered D(1) dopamine receptor trafficking in parkinsonian and dyskinetic non-human primates. Neurobiol Dis. 2007;26:452–463. 17350277 10.1016/j.nbd.2007.02.001

[92] Rinne UK, Laihinen A, Rinne JO, Nagren K, Bergman J, Ruotsalainen U. Positron emission tomography demonstrates dopamine D2 receptor supersensitivity in the striatum of patients with early Parkinson’s disease. Mov Disord. 1990;5:55–59. 2136932 10.1002/mds.870050114

[93] Lee T, Seeman P, Rajput A, Farley IJ, Hornykiewicz O. Receptor basis for dopaminergic supersensitivity in Parkinson’s disease. Nature. 1978;273:59–61. 692671 10.1038/273059a0

[94] Taverna S, Ilijic E, Surmeier DJ. Recurrent collateral connections of striatal medium spiny neurons are disrupted in models of Parkinson’s disease. J Neurosci. 2008;28:5504–5512. 18495884 10.1523/JNEUROSCI.5493-07.2008 PMC3235738

[95] Twedell EL, Bair-Marshall CJ, Girasole AE, Scaria LK, Sridhar S, Nelson AB. Striatal lateral inhibition regulates action selection in a mouse model of levodopa-induced dyskinesia. *bioRxiv*. [Preprint]. 10.1101/2024.10.11.617939

[96] Chernoloz O, El Mansari M, Blier P. Sustained administration of pramipexole modifies the spontaneous firing of dopamine, norepinephrine, and serotonin neurons in the rat brain. Neuropsychopharmacology. 2009;34:651–661. 18688211 10.1038/npp.2008.114

[97] Ray NJ, Miyasaki JM, Zurowski M, et al Extrastriatal dopaminergic abnormalities of DA homeostasis in Parkinson’s patients with medication-induced pathological gambling: A [11C] FLB-457 and PET study. Neurobiol Dis. 2012;48:519–525. 22766031 10.1016/j.nbd.2012.06.021 PMC3465363

[98] Tokunaga N, Choudhury ME, Nishikawa N, et al Pramipexole upregulates dopamine receptor D(2) and D(3) expression in rat striatum. J Pharmacol Sci. 2012;120:133–137. 22986363 10.1254/jphs.12096sc

[99] Collins AG, Frank MJ. Opponent actor learning (OpAL): Modeling interactive effects of striatal dopamine on reinforcement learning and choice incentive. Psychol Rev. 2014;121:337–366. 25090423 10.1037/a0037015

[100] Mohebi A, Wei W, Pelattini L, Kim K, Berke JD. Dopamine transients follow a striatal gradient of reward time horizons. Nat Neurosci. 2024;27:737–746. 38321294 10.1038/s41593-023-01566-3 PMC11001583

[101] Mamad O, Delaville C, Benjelloun W, Benazzouz A. Dopaminergic control of the globus pallidus through activation of D2 receptors and its impact on the electrical activity of subthalamic nucleus and substantia nigra reticulata neurons. PLoS One. 2015;10:e0119152. 25742005 10.1371/journal.pone.0119152 PMC4350999

[102] Rommelfanger KS, Wichmann T. Extrastriatal dopaminergic circuits of the basal ganglia. Front Neuroanat. 2010;4:139. 21103009 10.3389/fnana.2010.00139 PMC2987554

[103] Surmeier DJ, Song WJ, Yan Z. Coordinated expression of dopamine receptors in neostriatal medium spiny neurons. J Neurosci. 1996;16:6579–6591. 8815934 10.1523/JNEUROSCI.16-20-06579.1996 PMC6578920

