# Supplementary material for: Aberrant striatal firing mediates impulsive decision-making in a mouse model of Parkinson's disease
Source: Brain. 2025 Sep 4;149(1):238–51. doi: 10.1093/brain/awaf312 (PMC12597312; doi:10.1093/brain/awaf312)
Supplement: awaf312_Supplementary_Data [file awaf312_supplementary_data.pdf]

Supplementary material

Aberrant Striatal Firing Mediates Impulsive Decision-Making in a Mouse Model of Parkinson's Disease

Xiaowen Zhuang,[1,2,3,4] Julia Lemak,[1,2,3,4] Sadhana Sridhar[1,2,3,4] and Alexandra B. Nelson[1,2,3,4]

Author affiliations:

1 Kavli Institute for Fundamental Neuroscience, UCSF, San Francisco, CA 94158, USA

2 Weill Institute for Neurosciences, UCSF, San Francisco, CA 94158, USA

3 Department of Neurology, UCSF, San Francisco, CA 94158, USA

4 Aligning Science Across Parkinson's (ASAP) Collaborative Research Network, Chevy Chase, MD, 20815, USA

Correspondence to: Alexandra B. Nelson

UCSF MC 0663, 675 Nelson Rising Lane, San Francisco, CA 94158, USA

E-mail: Alexandra.nelson@ucsf.edu

The Supplementary material includes:

1. FIGURES & LEGENDS for Fig. S1 to S8
2. MATERIALS AND METHODS
    - Animals
    - Surgical Procedures
    - Motor assessment
    - Operant Training and Assessment
    - *In vivo* Electrophysiology
    - *Ex vivo* Electrophysiology
    - Histology & Quantification
    - Statistical analysis
    - Experimental design & Statistical analysis
3. AUTHOR CONTRIBUTIONS
4. Table 1. EXPERIMENTAL ANALYSIS AND STATISTICS

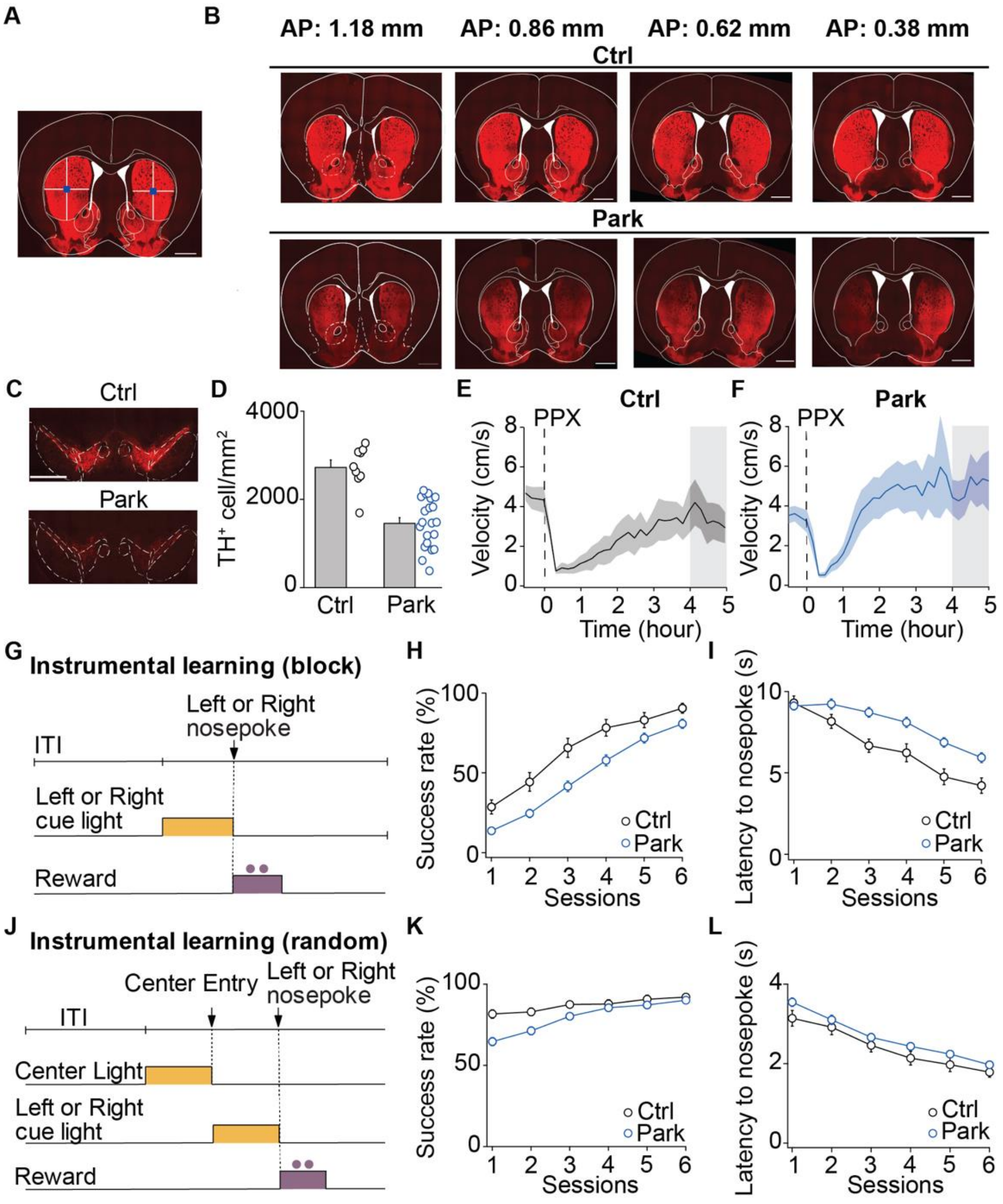

Figure S1 (Associated with Figure 1).
(**A-D**) Postmortem coronal sections were immunostained for tyrosine hydroxylase (TH, red). (A) Coronal section showing the division of the striatum into four quadrants. Two intersecting midlines, crossing at their midpoint (blue dot). (**B**) Representative striatal sections from saline-injected (control, top) and 6-OHDA-injected (parkinsonian, bottom) mice, spanning from +1.18 to +0.38mm anterior to bregma. (**C**) Representative midbrain sections containing the substantia nigra pars compacta (SNc) from control (top) and parkinsonian (bottom) mice. (**D**) Density of TH^{+} SNc dopamine neurons, normalized to the measured area (Ctrl: N = 9, Park: N = 21, $p < 0.001$). (**E,F**). Open field locomotor activity in control (E) and parkinsonian (F) mice following PPX injection. Operant behavior was tested during the shaded period (4-5 hours post-PPX) (E, N = 7, p = 0.074; F, N = 15, p = 0.02). **(G-L)** Detailed metrics from behavioral shaping (instrumental learning), in the absence of pramipexole treatment. (**G**) The instrumental learning (blocked) task structure, in which the same side port was cued in blocks. (**H**) The success rate (nosepoke at the cued side) increased in both healthy and parkinsonian mice across instrumental learning sessions (Ctrl: N = 34, Park: N = 81; session 1, 3&4: $p < 0.05$, session 2, 5&6: $p > 0.05$). (**I**) Average time between side cue light illumination and side nosepoke in instrumental learning sessions (session 1-2&6, $p > 0.05$; session 3-5, $p < 0.05$). (**J**) After blocked instrumental learning, animals were transition to the instrumental learning (random) task, in which animals initiated a trial with a center nosepoke, after which either side port could be cued on different trials within a block. (**K**) The success rate (trial self-initiation followed by cued side nosepoke) was high across instrumental learning sessions (Ctrl: N = 34, Park: N = 81; session 1-6: $p > 0.05$). (**L)** Average time between central cue light illumination and trial initiation (session 1-6: $p > 0.05$). N, animals, all data presented as means ± SEMs. Scale bars, 1mm.

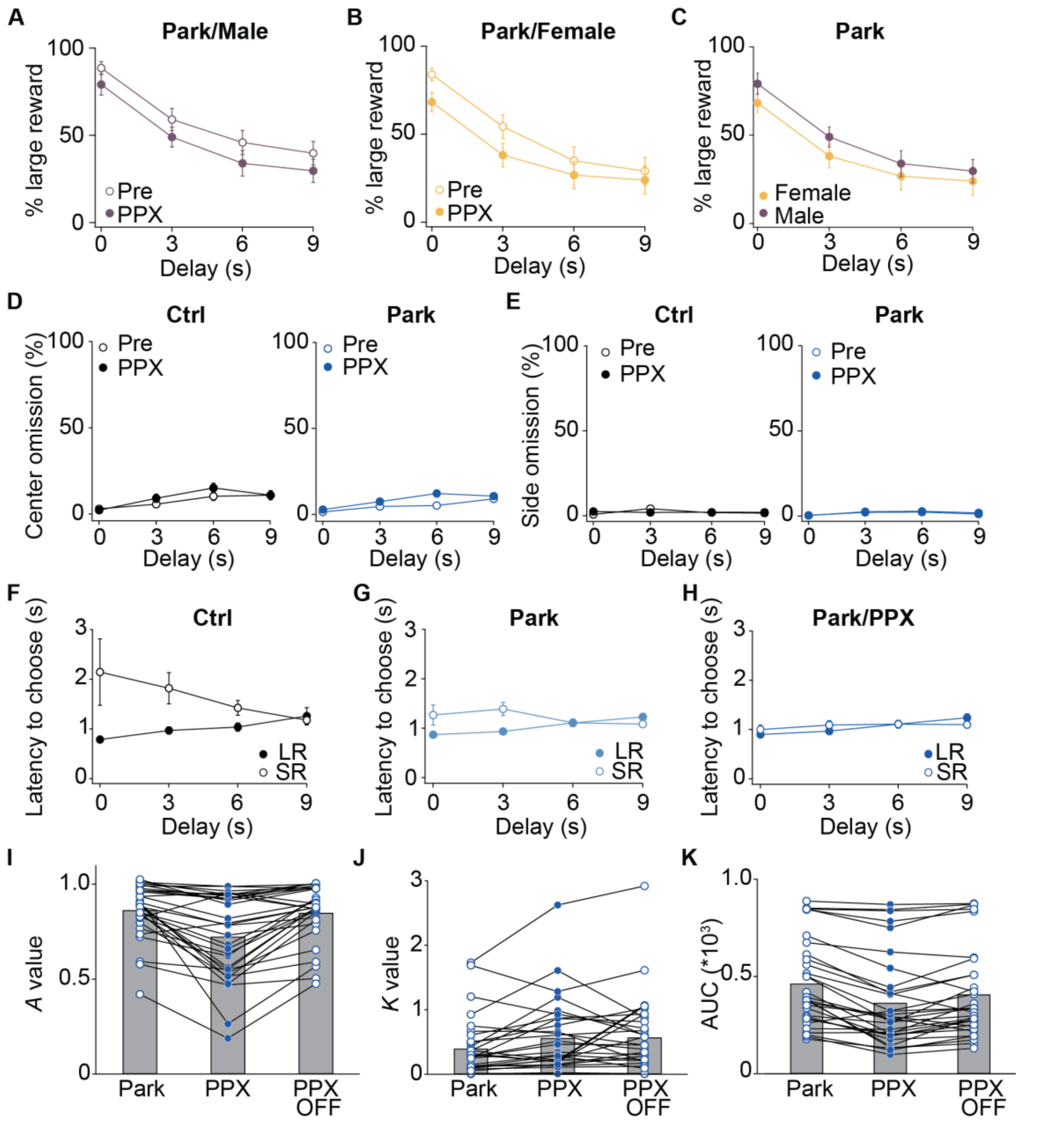

Figure S2 (Associated with Figure 1).

(**A-C**) Comparison of delay discounting behavior by sex. Percentage of trials in which male (A), female (B), or male vs. female (C) mice chose the delayed/large reward across delays during baseline (open circles) and following PPX injections (filled circles) (Male: N = 12, Female: N = 19; A, 0s: $p = 0.026$, 3s: $p = 0.014$, 6s: $p = 0.003$, 9s: $p = 0.015$; B, 0s: $p < 0.0001$, 3s: $p < 0.0001$, 6s: $p = 0.03$, 9s: $p = 0.366$; C, $p > 0.99$ at all delays. (**D**) Percentage of free choice trials in which mice did not initiate the trial (center omission, Ctrl: N = 16, Park: N = 31; Ctrl: $p > 0.05$ at all delays; Park: $p > 0.05$, except 6s: $p < 0.05$). (**E**) Percentage of free choice trials in which mice did not make a side (choice) nosepoke (side omission, Ctrl: N = 16, Park: N = 31; Ctrl: $p > 0.05$ at all delays; Park: $p > 0.05$ at all delays). (**F-H**) Time between cue light and choice nosepoke for delayed/large reward trials (filled circles) and immediate/small reward trials (open circles) at each delay during free choice trials in healthy control (F), parkinsonian (G) and PPX-treated parkinsonian mice (H) (Ctrl: N = 16, Park: N = 31; LR vs. SR, F, 0s: $p = 0.12$, 3s: $p < 0.01$, 6s: $p < 0.05$, 9s: $p > 0.99$; G, 0s: $p = 0.77$, 3s: $p < 0.05$, 6s: $p > 0.99$, 9s: $p = 0.56$; H, $p > 0.99$ at all delays). (**I-K**) *A*, *K* and AUC values in all three conditions in parkinsonian mice: baseline (Park), 4-5 hours after PPX treatment (PPX), and PPX OFF (48 hours washout) (N = 31, Park vs. PPX off, I: $p > 0.99$; J: $p = 0.01$; K: $p < 0.01$).

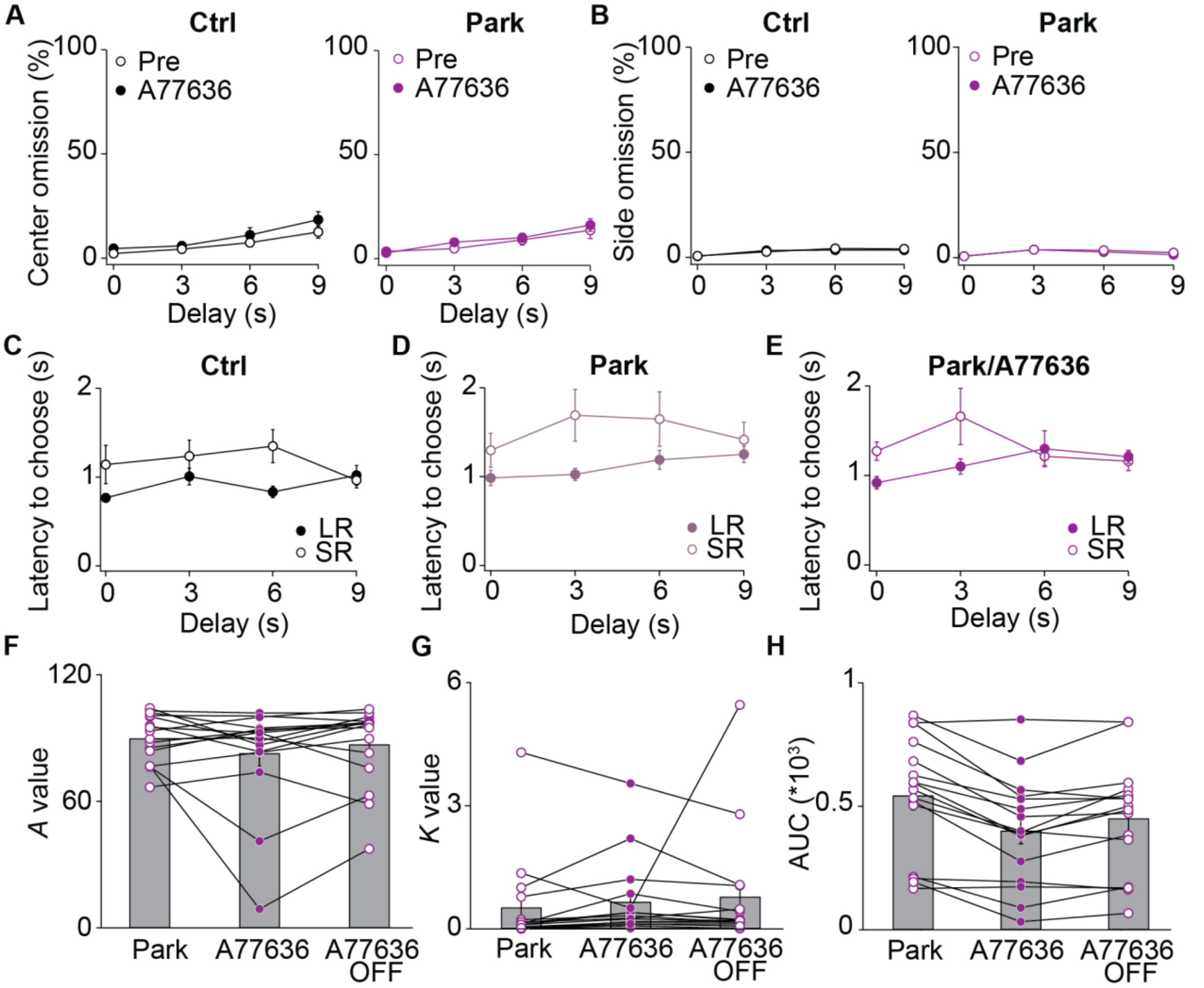

Figure S3 (Associated with Figure 2)
(A) Percentage of free choice trials in which mice did not initiate the trial (Ctrl: N = 10, Park: N = 16; Ctrl: $p > 0.05$ at all delays; Park: $p > 0.05$ at all delays). (**B**) Percentage of free choice trials in which mice did not make a side (choice) nosepoke, Ctrl: N = 10, Park: N = 16; Ctrl: $p > 0.05$ at all delays; Park: $p > 0.05$ at all delays). (**C-E**) Time between cue lights and choice nosepoke for delayed/large reward trials (filled circles) and immediate/small reward trials (open circles) at each delay during free choice trials in healthy control (C), parkinsonian (D) and A77636-treated parkinsonian mice (E); Ctrl: N = 10, Park: N = 16; LR vs. SR, C, 0s: $p = 0.17$, 3s: $p = 0.79$, 6s: $p < 0.05$, 9s: $p > 0.99$; D, 0s: $p = 0.75$, 3s: $p < 0.05$, 6s: $p = 0.20$, 9s: $p > 0.99$; E, $p > 0.05$ at all delays. (**F-H**) *A*, *K* and AUC values in all three conditions in parkinsonian mice: baseline, post-A77636 treatment, and A77636 washout (48 hours) (N = 16, A77636 vs. A77636 off, F: $p = 0.43$; G: $p = 0.96$; H: $p = 0.43$).

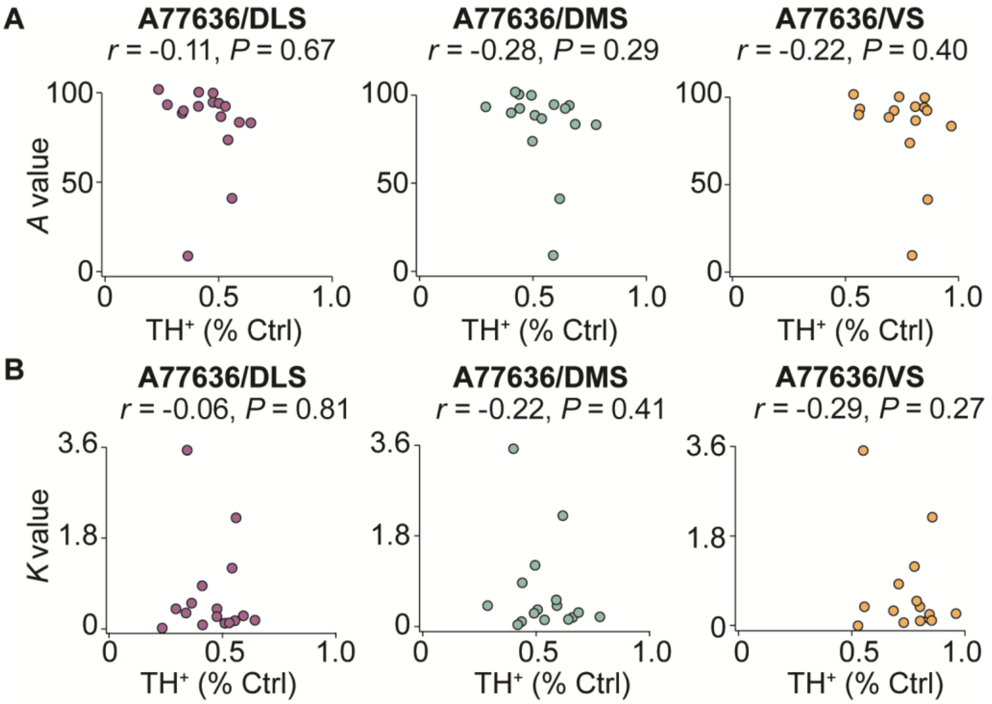

Figure. S4 (Associated with Figure. 3)

(A) Scatter plots of *A* values from delay discounting behavior in parkinsonian mice treated with A77636 versus residual striatal TH^+ fluorescence intensity, across subregions (N = 16) (**B**) Scatter plots of *K* values in parkinsonian mice treated with A77636 and residual striatal TH^+ fluorescence intensity, across subregions (N = 16). N = animals.

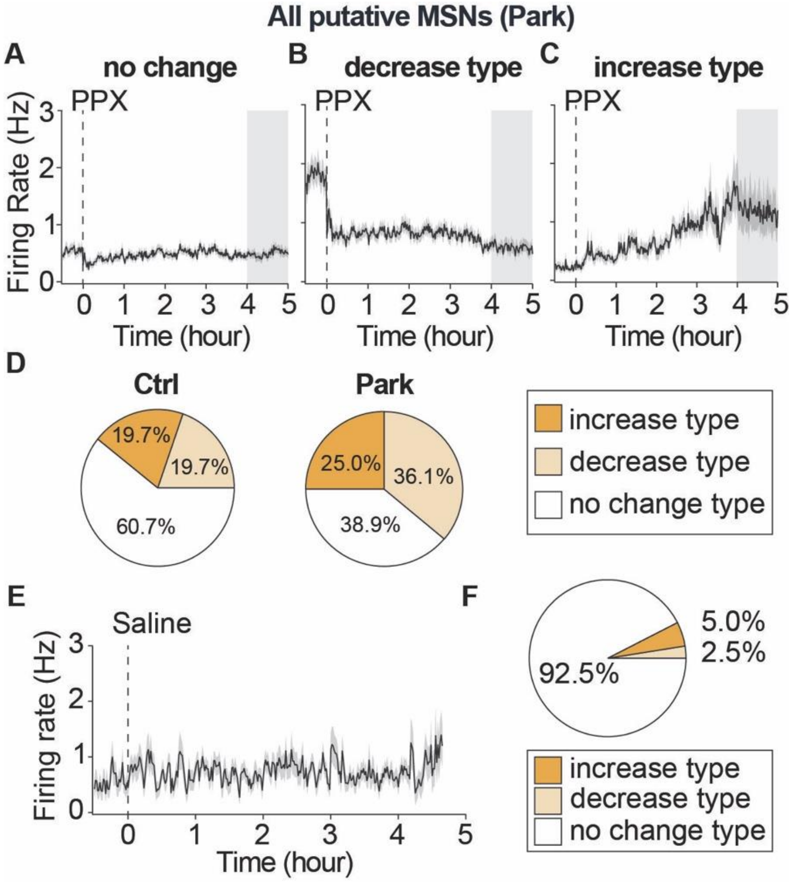

Figure S5 (Associated with Figure 4)
(**A-C**). Average firing rates of putative MSNs from 6-OHDA-treated (parkinsonian) mice, categorized based on response to PPX injection, measured as the difference between the average rates during the baseline and 4-5 hours after PPX injection (shaded block). Dotted line: PPX injection. Neurons were classified as 'increase' (A), 'decrease' (B), or 'no change' (C) (increase: n = 54, decrease: n = 78, no change: n = 82; N = 11). (**D**). Proportion of putative MSNs showing each PPX response type in saline-treated (control) (left: Ctrl, n = 117, N = 7) vs. parkinsonian mice (right: Park, n = 214, N = 11, p = 0.0003). (**E**). Average firing rate of putative MSNs from parkinsonian mice in response to saline administration (dotted line: saline i.p. injection, n = 40; N = 4). (**F**). Proportion of putative MSNs showing each Saline response type (increase: n = 1, decrease: n = 2, no change: n = 37), N, animals, n, cells. All data presented as means ± SEMs.

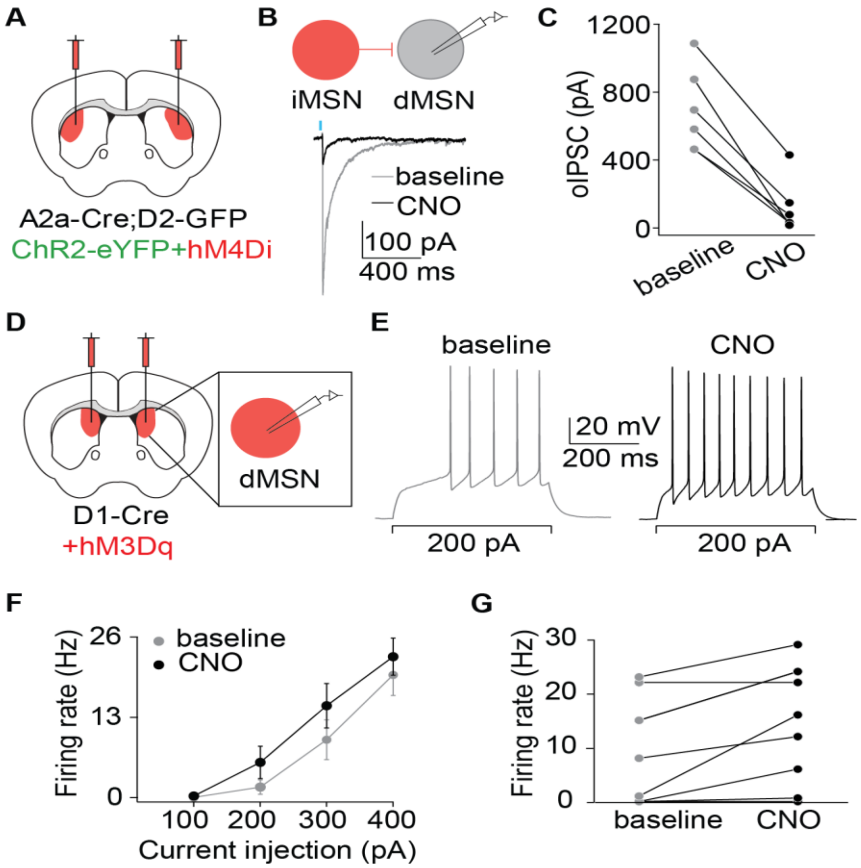

Figure S6 (Associated with Figure 5).
(**A-C**) The Gi DREADD construct was validated with *ex vivo* electrophysiology. (A) Cartoon of approach. A2a-Cre;D2-GFP mice were bilaterally injected with a combination of DIO-ChR2-eYFP and DIO-hM4D(Gi) into the DLS, and later sacrificed for *ex vivo* (slice) electrophysiology. (B) Top: cartoon of the recording configuration, in which eYFP-negative dMSNs were targeted for recording of optically-evoked local inhibitory inputs from iMSNs. Bottom: representative voltage-clamp recordings showing optically-evoked inhibitory postsynaptic potentials oIPSC before (black) and after (grey) bath application of CNO (1 µM). (C) Summary oIPSC amplitudes before and after bath application of CNO ($n = 6$, $N = 2$; $p < 0.01$). (**D-G**) The Gq DREADD construct was validated with *ex vivo* electrophysiology. (D) Cartoon of approach. D1-Cre mice were bilaterally injected with DIO-hM3D(Gq) into the DMS and later sacrificed for slice electrophysiology. (**E**) Representative current-clamp traces of action potential responses to current injection, at baseline and after CNO application, in a hM3Dq-expressing dMSN. (**F**) Summary of input-output curves in dMSNs before and after bath application of CNO. (**G**) Summary of responses of dMSNs to 300pA injection before and after CNO bath-application $n = 9$, $N = 3$; $p = 0.01$). N, animals, n, cells. All data presented as means ± SEMs. This slice validation data in A-C was also used in another manuscript.[1]

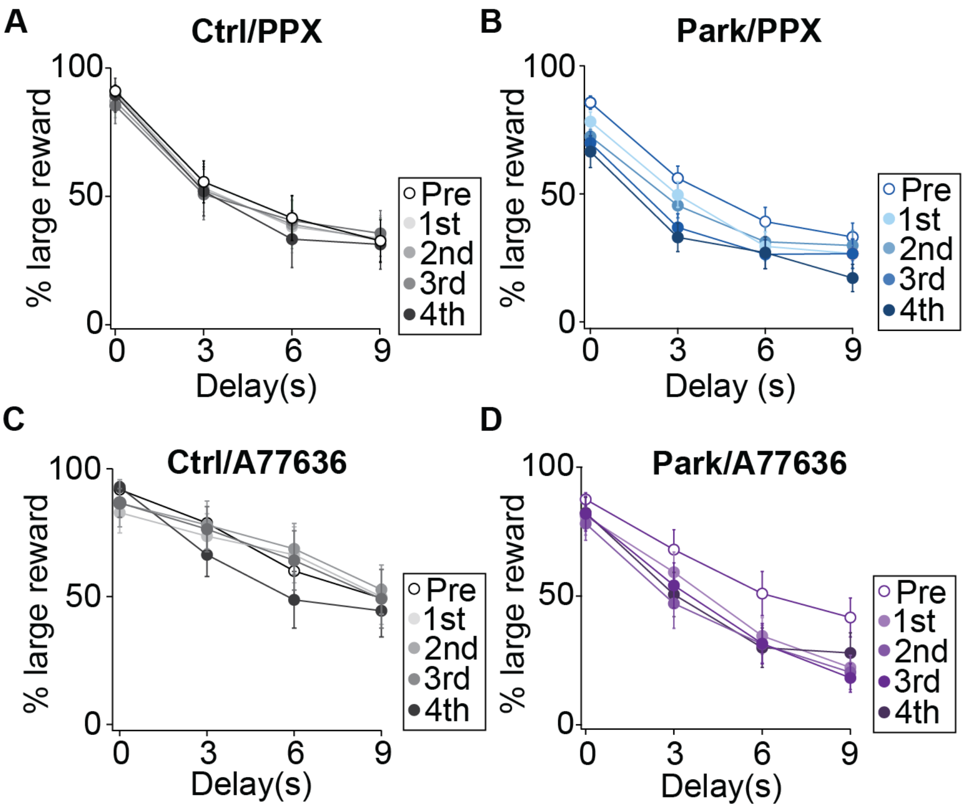

Figure S7 (Associated with Figure 6).
Delay discounting behavior shown across consecutive sessions with either the dopamine D2/3 agonist PPX (A,B) or the D1 agonist A77636 (C,D)**.** In each panel, the percentage of trials in which the delayed/large reward was chosen is plotted across delays. **(A,B)** Delay discounting behavior during baseline (open circles) and the 1st through 4th PPX sessions in healthy control (A) or parkinsonian (B) mice. **(C,D)** Delay discounting behavior during baseline (open circles) and the 1st through 4th A77636 sessions in healthy control (C) or parkinsonian (D) mice. A: N = 16, B: N = 31, C: N = 10, D: N = 16**.** N, animals, all data presented as means ± SEMs.

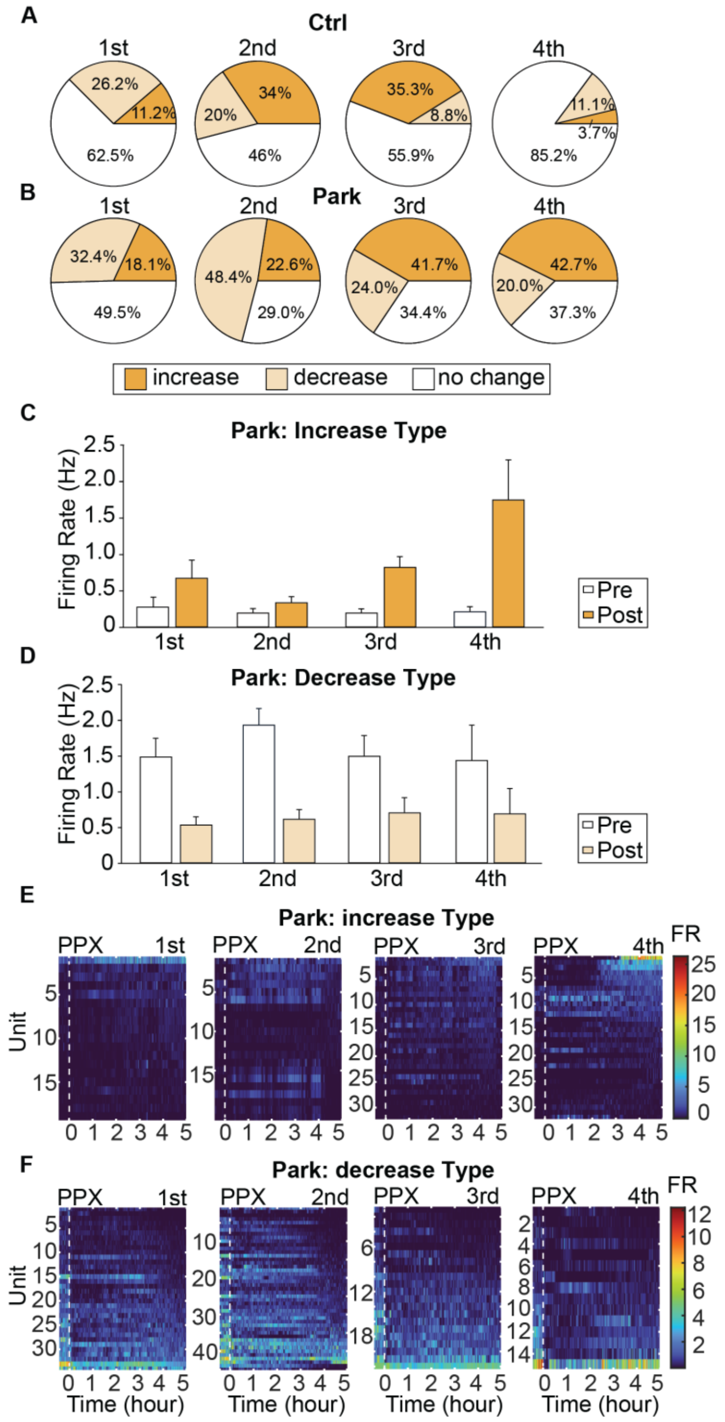

Figure S8 (Associated with Figure 6).
(**A-B**) Proportion of putative MSNs with an increase, decrease, or no change in firing rate in response to PPX (assessed 4 hours post-injection) in control (A) or parkinsonian (B) mice. (**C-D**) Average firing rates of 'increase' (C) or 'decrease' type (D) MSNs pre-PPX and across four post-PPX epochs in parkinsonian mice. (**E-F**). Heatmaps showing firing rates over time during 4 PPX injection sessions. Responses during the four sessions, are from left to right, for neurons with an increase (E) or decrease (F) type response. Each row represents a single unit. C&E: 1st: N = 6, n = 19. 2nd: N = 6, n = 21, 3rd: N = 7, n = 41, 4th: N = 7, n = 32; D&F: 1st: N = 9, n = 34. 2nd: N = 8, n = 45, 3rd: N = 7, n = 22, 4th: N = 5, n = 15, N, animals, n, cells. All data presented as means ± SEMs.

Materials and methods

Animals

Hemizygous BAC transgenic mice expressing Cre recombinase under the control of the Drd1a (D1-Cre, GENSAT BAC transgenic EY217) or Adora2a (A2a-Cre, GENSAT BAC transgenic KG139) regulatory elements were used to restrict the expression of Cre-dependent constructs to direct and indirect medium spiny neurons, dMSNs and iMSNs, respectively. For *ex vivo* slice physiology experiments, A2a-Cre mice were bred to hemizygous Drd2-GFP mice, to generate A2a-Cre;Drd2-GFP mice. For operant behavior, mice were placed on food restriction 3 days before training, which was maintained during training and testing phases. Their weight was monitored closely to ensure that their food-restricted body weight remained around 85%-90% of their free-feeding body weight.

Surgical Procedures

For subsequent optically identified single-unit recordings, AAV5-DIO-ChR2-eYFP (UPenn Vector Core, 1.0 µL, undiluted) was injected into the left DMS (+ 0.8 mm AP, + 1.5 mm ML, – 2.5 mm DV) in a subset of mice. For chemogenetic experiments, AAV5-DIO-hM4D(G_i)-mCherry (UNC Vector Core, 1.0 µL, diluted 1:2 in sterile saline), AAV5-DIO-hM3q(G_q)-mCherry (UNC Vector Core, 1.0 µL, undiluted), or AAV5-DIO-mCherry (UNC Vector Core, 1.0 µL, undiluted) was injected in bilateral DMS (+ 0.8 mm AP, ± 1.5 mm ML, – 2.5 mm DV). Both 6-OHDA and virus were injected at a rate of 0.2 µL/min, after which the injection cannula was left in place for 10 min prior to being withdrawn, and the scalp being sutured. In preparation for *in vivo* single-unit recordings, D1-Cre and A2a-Cre mice were injected bilaterally with DLS 6-OHDA (or saline) and DMS DIO-ChR2-eYFP, followed by implantation of DMS optrode arrays in a second surgical procedure.

Motor Assessment

Each rotarod session included three trials with a 10-min intertrial interval. The rotation speed gradually increased from 5 to 80 RPM over 5 min. Mice were scored for their latency to fall

and the average across three trials was reported. Open field locomotion was also performed in a subset of mice to examine the therapeutic effect of PPX or A77636. Mice were habituated to the open field (clear acrylic cylinders, 25 cm in diameter) for 20 min, 1-2 days prior to behavioral sessions. During experimental sessions, overall movement was monitored with an overhead camera and analyzed offline using video-tracking software (Noldus Ethovision), including distance traveled and velocity.

Operant Training and Assessment

Operant behavior was conducted in nine custom-made 18 x 18 x 26 cm operant chambers, enclosed within sound-attenuating cabinets (Coulbourn Instruments and Med Associates). One side of each chamber was equipped with one yellow LED as a house light and remained illuminated during all experimental stages, unless stated otherwise. The opposite side of the chamber was equipped with left and right side nosepokes 12 cm apart. Each nosepoke contained two yellow LED stimulus lights: one positioned 6 cm above and another located inside the nosepoke. Sweetened condensed milk (diluted 1:3 with water) was delivered to a liquid receptacle from the central port, equidistant from the left and right nosepokes through a solenoid valve (The Lee Company). An infrared detector was mounted horizontally across the center port to detect head entries. Experimental events and data collection were managed by a PC running Arduino software. The Arduino scripts for running training and assessment phases are available here: https://zenodo.org/doi/10.5281/zenodo.10703131.

In Phase 1 (magazine training), which consisted of a single session, food-restricted mice were placed in the operant box, and liquid reward (10 μL of milk) was delivered on a random interval schedule of 40-80 s for a total of 40 rewards. Each trial began with the delivery of milk into the center port, accompanied by a 10 s illumination of the center port light.

In Phase 2 (blocked instrumental learning), mice were trained to nosepoke at the side ports to obtain reward on a fixed-ratio 1 (FR1) schedule. Each training session was separated into two blocks, separated by a two-minute break period when the house light was turned off. Each block lasted 50 min. Only one nosepoke (either the left or the right side) was trained in each block, and the active nosepoke switched during the break period. The initial nosepoke (left or right) was counterbalanced across sessions. Each trial began with a stimulus cue light above the left or right nosepoke being illuminated. If the mouse nosepoked that port within 20 s, the cue light was

extinguished, and reward (10 µL of milk) was delivered to the center port, which was illuminated for 10 s, followed by a random 30-50 s inter-trial interval. If the mouse did not perform a nosepoke on the correct side, then cue lights were extinguished and went into a timeout (a random 30-50 s interval). Mice were trained in Phase 2 for at least 6 sessions and until they met criteria (correct response on > 80% of trials). Mice that did not meet criteria after 10 sessions were removed from the study. Overall, 8 out of 125 mice were excluded during Phase 2.

Phase 3 (randomized instrumental learning) resembled Phase 2 with two additions: the introduction of self-initiated trial start, and pseudorandomized (no more than two consecutive trials with the same side cued), rather than blocked trials. Trials began with the center port being illuminated for 10 s. During this period, a trial could be initiated by a center port nosepoke. Failure to make a center nosepoke within this period was considered a center omission and was followed by a timeout period (random 30-50 s interval). Following an initiation nosepoke, the stimulus cue lights at either the left or right port were activated for 10 s. If the mouse nosepoked that port within 10 s, the cue light was extinguished, and reward (10 µL of milk) was delivered to the center port, which was illuminated for 10 s, followed by a random 30-50 s inter-trial interval. Failure to poke the correct side port within 10 s was considered a side omission, and cue lights were extinguished until the next trial. Poking the opposite, uncued side was recorded as an incorrect response, but did not trigger punishment. Each session was 90 min. Mice were trained in Phase 3 for at least 5 sessions and until criteria were met. Criteria were (1) responding correctly in at least 80% of trials, and (2) incorrect responses were less than 15% of the total (to reduce side bias). Mice that did not meet criteria in 8 sessions were excluded. Overall, 5 out of 117 mice were excluded during Phase 3.

Phase 4 (delay discounting) was the final task. A detailed protocol for delay discounting can be found at (DOI: dx.doi.org/10.17504/protocols.io.4r3l22dqql1y/v1). The delay discounting task was composed of three trial types: forced choice delayed/large, forced choice immediate/small, and free choice. Each session was composed of four blocks. Each block included 30 trials, starting with five forced choice delayed/large trials, followed by five forced choice immediate/small trials to remind mice of the delay contingencies in effect for that block. These "refresher" trials were followed by 20 free choice trials. In a given mouse, the left and right sides were assigned to either delayed/large or immediate/small outcomes, but the side assignments were randomly distributed between mice. Each trial was a fixed duration of 50 s. Therefore, port choice did not influence the

trial duration (i.e., choosing the small/immediate reward did not allow mice to proceed to the next trial more quickly). The delay preceding large reward delivery increased across a session (between blocks) from 0 s (block 1) to 3 s (block 2), to 6 s (block 3) to 9 s (block 4). After 2-3 weeks of training on the task, once mice had established a stable delay discounting curve, the delay discounting performance was evaluated in three states: "pre-treatment (Pre)", "pramipexole (PPX) or A77636 ON" (4h after PPX or 30min after A77636), and "OFF" (48h after PPX or A77636). Overall, 15 out of 112 mice were excluded in Phase 4 from the study because they were unable to establish stable delay discounting behavior within 3 weeks. All trials started with 10 s illumination of the center port light. A center nosepoke within 10 s extinguished the light and initiated the trial. Trials on which mice failed to nosepoke during this window were recorded as center omissions. During forced choice delayed/large trials, cue lights on one side were illuminated for 10 s, and nosepoke at that port resulted in a large reward (15 μL) delivered to the center port after a delay period, as described below. During forced choice immediate/small trials, cue lights on the other side were illuminated for 10 s, and nosepoke at that port resulted in a small (5 μL) reward delivered to the center port immediately. During free choice trials, both left and right cue lights were illuminated for 10 s. Nosepokes on either side resulted in reward, following the contingencies introduced during the "refresher" trials. If a mouse failed to nosepoke in one of the side ports within 10 s, cue lights were extinguished, and the trial was recorded as a side omission. After reward delivery or side omission, the inter-trial interval (adjusted to achieve trial start every 50s) began.

In vivo Electrophysiology

During each session, animals were plugged into a lightweight multiplexed and commutated headstage cable to record single-unit activity (CerePlex Direct, Blackrock Microsystems). Spike waveforms were filtered at 154-8800 Hz and digitized at 30 kHz. The experimenter manually set a threshold for storage of electrical events. Spike sorting and single units were identified offline by manual sorting into clusters (Offline Sorter, Plexon). Waveform features used for separating units were typically a combination of valley amplitude, the first three principal components (PCs), and/or nonlinear energy. Clusters were classified as single units if they fulfilled the following criteria: (1) the unit's waveforms were statistically different from multiunit activity and any other single units on the same wire, in 3D PCA space, (2) no inter-spike interval < 1 msec was observed.

Single-units were then classified as putative medium spiny neurons (MSNs) or interneurons as previously described using features of the spike waveform (peak to valley and peak width),[2,3] as well as inter-spike interval distribution. After single-units had been selected for further study, their firing activity was analyzed using NeuroExplorer 4.133 (Nex Technologies).

To determine if a unit was optogenetically identified, a peristimulus time histogram was constructed around the onset of laser pulses. To be considered optogenetically identified, a unit had to fulfill 3 criteria: (1) increase firing rate above the 99% confidence interval of the baseline within 15 msec of laser onset; (2) exceed this threshold for at least 15 msec; and (3) have laser-activated waveforms not statistically distinguishable from spontaneous waveforms.

Ex vivo Electrophysiology

A detailed protocol is available at (DOI: dx.doi.org/10.17504/protocols.io.b9uir6ue). Additional details are provided in the Supplementary material. Briefly, slice electrophysiology was used to validate the inhibitory and excitatory Designer Receptor Exclusively Activated by Designer Drug (DREADD) constructs for chemogenetic inhibition and excitation experiments. For chemogenetic inhibition experiments, acute slices from A2a-Cre;Drd2-GFP mice coinjected with AAV5-DIO-ChR2-eYFP (UPenn Vector Core) and AAV5-DIO-hM4D(Gi)-mCherry (UNC Vector Core) were prepared. To measure the acute effect of CNO on the synaptic output of iMSNs in slice, dMSNs were targeted for whole-cell recordings and identified by their GFP/eYFP-negative somata in striatal regions showing mCherry positive processes. For chemogenetic excitation experiments, acute slices from D1-Cre mice injected with AAV5-DIO-hM3q(G_q)-mCherry (UNC Vector Core) were prepared. To measure the acute effect of CNO on dMSN excitability in slice, mCherry-positive dMSNs were targeted for whole-cell recordings. Neurons with GABAergic interneuron physiological properties (membrane tau decay < 1 ms) were excluded from further analysis.

Mice were deeply anesthetized with ketamine-xylazine (100-200 mg, i.p.) and perfused with a carbogenated, ice-cold glycerol-based artificial cereberospinal fluid (ACSF) solution containing (in mM): 250 glycerol, 2.5 KCl, 1.2 NaH2PO4, 10 HEPES, 21 NaHCO3, 5 D-glucose, 2 MgCl2, 2 CaCl2. Following decapitation, brains were dissected, mounted on a chuck, and submerged in ice-cold glycerol solution. A vibrating microtome (Leica) was used to cut sequential 275 mm coronal slices containing the striatum, which were immediately transferred to warm

(34□C), carbogenated ACSF containing (in mM): 125 NaCl, 26 $NaHCO_3$, 2.5 KCl, 1.25 NaH_2PO_4, 12.5 D-glucose, 1 $MgCl_2$, 2 $CaCl_2$. Slices were incubated for 30-60 min, then kept at room temperature until use.

Whole-cell voltage-clamp and current-clamp recordings were made using a MultiClamp 700B amplifier (Molecular Devices) and ITC-18 A/D board (HEKA). Data was acquired using Igor Pro 6.0 software (Wavemetrics) and custom acquisition routines (mafPC, courtesy of M. A. Xu-Friedman). Recordings were filtered at 2 kHz and digitized at 10 kHz. The synaptic currents were recorded with a cesium methanesulfonate-based internal with high chloride, which contained (in mM): 120 CsCl, 15 $CsMESO_3$, 8 NaCl, 0.5 EGTA, 10 HEPES, pH = 7.3 and monitored at a holding potential of -70 mV. Series resistance and leak currents were monitored continuously. The intrinsic excitability was recorded with a potassium methanesulfonate-based internal, which contained (in mM): 130 $KMeSO_3$, 10 NaCl, 2 $MgCl_2$, 0.16 $CaCl_2$, 0.5 EGTA, 10 HEPES, 2 MgATP, 0.3 NaGTP, pH 7.3. Inhibitory synaptic currents were optically evoked using 3 ms pulses of 473 nm light ranging in power from 0.5-4 mW and delivered by a TTL-controlled LED (Olympus) passed through a GFP filter (Chroma). oIPSCs were elicited every 20 s and the amplitudes were compared before and 10-15min after addition of CNO to the ACSF (1 μM). Current-clamp recordings were made to measure the intrinsic excitability of striatal neurons. A series of square-wave current steps, ranging from 100pA to 400pA, in 100pA increments were delivered to obtain the input-output properties. Firing rates were compared before and 10-15min after addition of CNO to the ACSF (1 μM).

Pharmacology

6-OHDA (Sigma Aldrich) for striatal dopamine depletions was prepared at 2.5 μg/μL in sterile saline on the day of surgery and wrapped in aluminum foil until use. Pramipexole dihydrochloride (Sigma Aldrich) was prepared in sterile saline solution and administrated via i.p. injection at a dose of 0.5 mg/kg, at least four times during the delay discounting assessment stage. A77636 (Tocris Biosciences) was prepared in sterile saline solution and administrated via i.p. injection at a dose of 1 mg/kg, approximately four times during the delay discounting assessment stage. Cognitive or motor assessments were conducted approximately 4 h after PPX injection or 30 min after A77636 injection. At this time point, locomotion was increased (avoiding the initial period of reduced locomotion).[4] Clozapine-N-oxide (CNO, Tocris Bioscience) was dissolved in

sterile saline, and injected i.p. at a final dose of 3 mg/kg for chemogenetic inhibition and 1mg/kg for chemogenetic excitation experiments. These CNO concentrations were chosen as it increased locomotion in parkinsonian mice without disrupting the cognitive assessment. The stock solution of CNO was wrapped in aluminum foil to minimize light exposure. For *ex vivo* experiments, CNO was dissolved in sterile saline and then diluted in ACSF for a final concentration of 1 μM.

Histology, Quantification and Microscopy

Following *in vivo* electrophysiology experiments, prior to perfusion, electrode array location was marked by electrolytic lesioning. After deep anesthesia, the implant was connected to a solid state, direct current (DC) Lesion Maker (Ugo Basile). A current of 100 μA was passed through each microwire for 5 s. To confirm dopamine depletion, tissue was immunostained for tyrosine hydroxylase (TH). Images at 10x magnification for striatal sections and 20x for SNc sections were acquired using a Nikon 6D conventional widefield microscope. The Mouse Brain Atlas in Stereotaxic Coordinates (hard copy, 4th edition) was consulted for anatomical reference. If postmortem studies showed evidence of no dopamine depletion or viral expression, their results were excluded from further analysis (N = 8 animals were excluded from the entire study; 5 based on no viral expression, and 3 based on no dopaminergic depletion).

Consistent lighting and exposure settings were maintained for all sections from both healthy and parkinsonian groups to allow for reliable comparison and subsequent image analysis. To quantify the residual TH positive fluorescence intensity within the striatum, 5-8 coronal sections between (AP 0.38 mm-1.18 mm) were analyzed. Regions of interest (ROIs) were outlined using the freehand ROI tool in ImageJ based on anatomical landmarks for striatum. The ROIs included 4 subregions: dorsolateral striatum (DLS), dorsomedial striatum (DMS), ventrolateral striatum (VLS), and ventromedial striatum (VMS). To divide the striatum into four subregions, the external boundary of the entire striatum was first traced using anatomical landmarks such as the external capsule and corpus callosum. Two midline boundaries were then established: a vertical midline at the geometric center of the width of the striatum, and a horizontal midline at the midpoint of its height. Two midlines were carefully aligned so that they intersected precisely at the midpoint of each other, forming a cross at the exact center of the striatum. This division resulted in four quadrants. VLS and VMS were then combined into ventral striatum (VS) for analysis. Subsequent analysis involved setting measurement parameters to include Area, Raw Integrated

Density, and Mean Gray Value. These parameters were measured for each ROI and 4 background areas, which showed minimal fluorescence to account for background signal. The mean background fluorescence was first calculated, followed by the calculation of corrected total fluorescence (CTF) using the formula: (Raw Integrated Density-(Area x Mean Fluorescence of background)) / Area of ROI. This process was repeated for both healthy and parkinsonian mice to calculated the normalized TH fluorescence levels.

To quantify the residual dopaminergic neurons within the midbrain, the substantia nigra pars compacta (SNc) region was outlined using the Allen Brain Atlas as a reference. TH-positive cells were manually counted per section using the point tool function in ImageJ. Counting was performed bilaterally and blinded to experimental condition across 4 stained sections per mouse, (AP -3.07 mm, -3.15 mm, -3.39 mm and -3.51 mm). Dopaminergic cell density was calculated and compared between healthy and parkinsonian conditions.

Statistical Analysis

The effects of drugs (PPX or A77636) or chemogenetic manipulations on delayed/large reward choice, omissions, trigger latency were compared within subjects using two-way repeated measures ANOVA followed by Bonferroni multiple comparisons. The effects of drugs (PPX or A77636) or chemogenetic manipulations on other parameters (K, A and AUC) of delay discounting performance were compared within subjects using Friedman repeated measures with post-hoc Dunn's multiple comparisons.

EXPERIMENTAL DESIGN & STATISTICAL ANALYSIS

Behavior

For analysis of delay discounting performance, the primary outcome was the percentage of large reward choices at each delay. Secondary outcome measures included other parameters (detailed below) that characterize overall delay discounting behavior during free choice trials. Raw data files were analyzed using custom analysis code written in Python (https://zenodo.org/doi/10.5281/zenodo.10703139). This code extracted specific task-related events, including trial start, cue light start, left and right nosepokes, reward consumptions and

timeouts during both forced and free choice trials. Choice performance in each block of the delay discounting task was measured as the percentage of free choice trials (excluding omissions) on which mice chose the delayed/large reward. Each mouse was trained on the delay discounting task until it reached stable baseline performance. Stable baseline was first assessed in each mouse by visually inspecting discounting curves for three sessions, then by calculation of the coefficient of variation for choice of the delayed/large outcome (stable defined as CV < 20% in at least 3 blocks for three consecutive sessions, or CV < 20% in each block for two consecutive sessions). Once performance was stable across sessions, delay discounting behavior was tested in the following conditions: baseline (pretreatment); PPX ON (4 h following the 1st, 2nd, 3rd, and 4th PPX injections); and PPX OFF (48 h after each PPX injection, with a saline injection given instead). Latencies to choose delayed/large rewards and immediate/small rewards during free choice trials were measured as the interval between the illumination of the nosepoke (side) cue light and a nosepoke (side) response, excluding side omissions. The effect of PPX on the delay discounting performance, including delayed/large reward choice, latency to choose, and omissions, at different delays, were determined using two-way repeated measures ANOVA, followed by Bonferroni multiple comparisons between baseline and PPX ON. To measure the chronic effects of PPX on the choice of delayed/large reward during delay discounting task, the 1st and 4th PPX injections were compared across different delays using two-way repeated measures ANOVA, followed by Bonferroni multiple comparisons between baseline and PPX ON or PPX OFF conditions.

To better understand the effects of PPX on delay discounting performance in which PPX significantly favored small/immediate choice in parkinsonian mice, additional analyses were conducted to capture how PPX specifically altered the shape of the discounting curve. The data was fitted to Herrnstein's simple hyperbolic model: $V = A/(1+KD)$ to estimate the discount rate parameter (K), reward size parameter (A), and the area under the fitted curve (AUC).[34, 41] Effects of PPX on these parameters in parkinsonian group were analyzed using Friedman repeated measures with post-hoc Dunn's multiple comparisons between baseline and PPX ON or OFF conditions.

For assessing motor ability, the primary outcome measures were the latency to fall (accelerating rotarod test) and average velocity (open field test), compared between healthy and parkinsonian mice using the Mann-Whitney U test test. The therapeutic effects of PPX on rotarod performance and velocity (240-300 min after PPX) were determined using the Wilcoxon Signed-

Rank test. The therapeutic effects of A77636 on open field locomotion (30-100 min after A77636) were determined using the Wilcoxon Signed-Rank test.

In Vivo Electrophysiology

In vivo electrophysiology sessions lasted 330 min. Firing rates were averaged in 1 minute bins. The modulation of firing rate by PPX was determined by comparing average firing rate before (-30-0 min) and after (240-300 min) PPX administration. This epoch matched the timing of the cognitive assessments after PPX. The 30-min baseline period was compared to two 30-min periods following drug injection (241-270 min and 271-300 min post-injection). Both optically labeled and unidentified MSNs were categorized into three groups as follows, based on the direction of change in firing rate according to Wilcoxon Signed-Rank test ($p < 0.01$): increase type (significant increase in both 30-min post injection periods), decrease type (significant decrease in both 30-min post injection periods) or no change units (no significant change in either of 30-min post injection periods).

Fisher's exact test was performed to compare the percentages of the three response types (increase, decrease and no change) between the 1st and 4th PPX injections in both control and parkinsonian groups. Firing rates of optogenetically labeled dMSNs and iMSNs recorded from parkinsonian mice were compared to recordings from healthy mice using the Mann-Whitney U test. Firing rates of optically-identified MSNs in parkinsonian mice before and after PPX administration were compared using the Wilcoxon Signed-Rank test.

Chemogenetic manipulation

In inhibitory DREADD validation slice electrophysiological experiments, oIPSC amplitudes were compared before and 10-15 min after addition of CNO, using the Wilcoxon Signed-Rank test. The effect of chemogenetic inhibition on gross locomotion was assessed by comparing average movement velocity during baseline (-20-0 min before CNO) and 20-30 min following CNO administration, using the Wilcoxon Signed-Rank test.

In excitatory DREADD validation slice electrophysiological experiments, firing rates were compared before and 10-15 min after addition of CNO, using Wilcoxon Signed-Rank test.

In chemogenetic manipulations during the delay discounting task, performance was compared within subjects in pre-CNO treatment periods versus 30 min post-CNO administration. Similar to pharmacological administration experiments, a stable baseline was established across at least three consecutive sessions. Post CNO administration sessions were averaged per animal across four CNO treatment sessions. Performance in free choice delay discounting trials across different delays were compared using two-way repeated measures ANOVA, followed by Bonferroni multiple comparisons. Other delay discounting parameters (*A*, *K* and AUC) were also compared in pre- and post-CNO sessions using Friedman repeated measures with post-hoc Dunn's multiple comparisons

Quantitative Histology

The relationship between dopamine depletion and delay discounting behavior parameters (*A*, *K* and AUC*)* after pharmacological administration (PPX or A77636) was quantified with Pearson correlation. Correlation coefficient (r) and p values were used to describe the linear correlation, and the coefficient of determination r^2 was used to describe the power of the model.

Author Contributions

XZ and ABN conceptualized the study. XZ, JL, and SS conducted experiments. XZ and JL analyzed the data. XZ developed analysis and acquisition code, curated data, created visualizations, and wrote the first draft of the paper. ABN supervised the project, provided resources and edited the paper.

References

1. Twedell EL, Bair-Marshall CJ, Girasole AE, Scaria LK, Sridhar S, Nelson AB. Striatal lateral inhibition regulates action selection in a mouse model of levodopa-induced dyskinesia. *bioRxiv*. Oct 12 2024;doi:10.1101/2024.10.11.617939

2. Berke JD, Okatan M, Skurski J, Eichenbaum HB. Oscillatory entrainment of striatal neurons in freely moving rats. *Neuron*. Sep 16 2004;43(6):883-96. doi:10.1016/j.neuron.2004.08.035

3. Gage GJ, Stoetzner CR, Wiltschko AB, Berke JD. Selective activation of striatal fast-spiking interneurons during choice execution. *Neuron*. Aug 12 2010;67(3):466-79. doi:10.1016/j.neuron.2010.06.034

4. Chang WL, Geyer MA, Buell MR, Weber M, Swerdlow NR. The effects of pramipexole on prepulse inhibition and locomotor activity in C57BL/6J mice. *Behav Pharmacol*. Mar 2010;21(2):135-43. doi:10.1097/FBP.0b013e328337be7e

Key Experiments	Figure	Comparison	Statistical test	N (animals)	n (units)	*p* value
**Table 1. EXPERIMENTAL ANALYSIS AND STATISTICS**<br>WSR, Wilcoxon Signed Rank test; MWU; Wilcoxon Rank Sum or Mann–Whitney U test; KW, Kruskal-Wallis test						
TH^+ fluorescence	Fig. 1B	Between group	KW<br>Dunn's	N = 44	NA	DMS vs. DLS: p = 0.09<br>DMS vs. VS: p = 0.0003<br>DLS vs. VS: p < 0.0001
PPX effects on rotarod	Fig. 1C	Between group	KW<br>Dunn's	Ctrl: N = 31<br>Park/PPX: N = 45<br>Park/Saline: N = 27	NA	Ctrl vs. Park/PPX: p < 0.001<br>Park vs. PPX: p < 0.001<br>Park vs. Saline: p = 0.95
DD curve during baseline (Ctrl vs. Park)	Fig.1 F&G	Between group	2way ANOVA, Bonferroni	Ctrl: N = 16<br>Park: N = 31	NA	Depletion: F(1, 180) = 3.14e-003, p = 0.9554, ηp² = 0.00001<br>delay: F(3, 180) = 38.53, p < 0.0001, ηp² = 0.3801<br>delays x Depletion F(3, 180) = 0.20, p = 0.8965, ηp² = 0.0020<br><br>0s: p > 0.99<br>3s: p > 0.99<br>6s: p > 0.99<br>9s: p > 0.99
PPX effects on DD curve (Ctrl)	Fig. 1F	Within animal	2way ANOVA, Bonferroni	N = 16	NA	PPX: F(1, 60) = 0.9564, p = 0.3320, ηp² = 0.015<br>delay: F(3, 60) = 16.06, p < 0.0001, ηp² = 0.347<br>delays x PPX: F(3, 60) = 0.4011, p = 0.7527, ηp² = 0.020<br><br>0s: p > 0.99<br>3s: p > 0.99<br>6s: p > 0.99<br>9s: p > 0.99
PPX effects on DD curve (Park)	Fig.1G	Within animal	2way ANOVA, Bonferroni	N = 31	NA	PPX: F(1, 120) = 95.48, p < 0.0001, ηp² = 0.027<br>delay: F(3, 120) = 22.36, p < 0.0001, ηp² = 0.336<br>delays x PPX: F(3, 120) = 2.05, p =

						0.1107, ηp^2 = 0.0017<br><br>0s: p < 0.0001<br>3s: p < 0.0001<br>6s: p = 0.0001<br>9s: p = 0.0087
PPX effects on *A* value (Park)	Fig. 1I	Within animal	Friedman, Dunn's	N = 31	NA	Park vs. PPX: p < 0.0001
PPX effects on *K* value (Park)	Fig. 1J	Within animal	Friedman, Dunn's	N = 31	NA	Park vs. PPX: p = 0.03
PPX effects on AUC value (Park)	Fig. 1K	Within animal	Friedman, Dunn's	N = 31	NA	Park vs. PPX: p < 0.0001
SNc cell density (Ctrl vs. Park)	Fig.S1D	Between group	MWU	Ctrl: N = 9<br>Park: N = 21	NA	P < 0.001
PPX effects on velocity (Ctrl)	Fig. S1E	Within animal	WSR	N = 8	NA	p = 0.074
PPX effects on velocity (Park)	Fig. S1F	Within animal	WSR	N = 15	NA	p = 0.02
Instrumental learning (block) (Ctrl vs. Park)	Fig.S1H	Between group	MWU, Bonferroni	Ctrl:<br>N = 34<br>Park:<br>N = 81	NA	session 1: p < 0.01<br>session 2: p = 0.06<br>session 3: p < 0.05<br>session 4: p < 0.01<br>session 5: p = 0.10<br>session 6: p = 0.09
Instrumental learning (block) Latency to nosepoke (Ctrl vs. Park)	Fig. S1I	Between group	MWU, Bonferroni	Ctrl:<br>N = 34<br>Park:<br>N = 81	NA	session 1: p >0.99<br>session 2: p >0.05<br>session 3: p < 0.001<br>session 4: p < 0.01<br>session 5: p < 0.01<br>session 6: p = 0.10
Instrumental learning (random) (Ctrl vs. Park)	Fig. S1K	Between group	MWU, Bonferroni	Ctrl:<br>N = 34<br>Park:<br>N = 81	NA	session 1: p = 0.56<br>session 2: p > 0.99<br>session 3: p > 0.99<br>session 4: p > 0.99<br>session 5: p = 0.75<br>session 6: p > 0.99
Instrumental learning (random) reaction time (Ctrl vs. Park)	Fig.S1L	Between group	MWU, Bonferroni	Ctrl:<br>N = 34<br>Park:<br>N = 81	NA	session 1: p = 0.38<br>session 2: p > 0.99<br>session 3: p > 0.99<br>session 4: p = 0.33<br>session 5: p = 0.16<br>session 6: p > 0.99

PPX effects on DD curve (Male)	Fig. S2A	Within animal	2way ANOVA, Bonferroni	N = 12	NA	PPX: $F(1, 44) = 39.98$, $p < 0.0001$, $\eta p^2 = 0.0328$<br>delay: $F(3, 44) = 13.35$, $p < 0.0001$, $\eta p^2 = 0.4436$<br>delays x PPX: $F(3, 44) = 0.11$, $p = 0.9516$, $\eta p^2 = 0.0003$<br><br>0s: $p = 0.026$<br>3s: $p = 0.014$<br>6s: $p = 0.003$<br>9s: $p = 0.015$
PPX effects on DD curve (Female)	Fig. S2B	Within animal	2way ANOVA, Bonferroni	N = 19	NA	PPX: $F(1, 72) = 57.63$, $p < 0.0001$, $\eta p^2 = 0.0251$<br>delay: $F(3, 72) = 11.22$, $p < 0.0001$, $\eta p^2 = 0.2991$<br>delays x PPX: $F(3, 72) = 3.45$, $p = 0.0208$, $\eta p^2 = 0.0045$<br><br>0s: $p < 0.0001$<br>3s: $p < 0.0001$<br>6s: $p = 0.030$<br>9s: $p = 0.366$
PPX effects on DD curve (Male vs. Female)	Fig. S2C	Within animal	2way ANOVA, Bonferroni	Male: N = 12<br>Female: N = 19	NA	Sex: $F(1, 116) = 2.87$, $p = 0.0931$, $\eta p^2 = 0.0166$<br>delay: $F(3, 116) = 17.47$, $p < 0.0001$, $\eta p^2 = 0.3029$<br>delays x PPX: $F(3, 116) = 0.07$, $p = 0.9782$, $\eta p^2 = 0.0011$<br><br>0s: $p > 0.99$<br>3s: $p > 0.99$<br>6s: $p > 0.99$<br>9s: $p > 0.99$
PPX effects on center omission (Ctrl)	Fig.S2D (left)	Within animal	2way ANOVA, Bonferroni	N = 16	NA	PPX: $F(1, 60) = 2.30$, $p = 0.1350$, $\eta p^2 = 0.0135$<br>delay: $F(3, 60) = 10.27$, $p < 0.0001$, $\eta p^2 = 0.2108$<br>delays x PPX: $F(3, 60) = 0.76$, $p = 0.5224$, $\eta p^2 = 0.0133$

						0s: p > 0.99<br>3s: p > 0.99<br>6s: p = 0.33<br>9s: p > 0.99
PPX effects on center omission(Park)	Fig.S2D (right)	Within animal	2way ANOVA, Bonferroni	N = 31	NA	PPX: F(1, 120) = 16.54, p < 0.0001, ηp² = 0.0300<br>delay: F(3, 120) = 8.58, p < 0.0001, ηp² = 0.1312<br>delays x PPX: F(3, 120) = 1.80, p = 0.1499, ηp² = 0.0098<br><br>0s: p > 0.99<br>3s: p = 0.15<br>6s: p = 0.0006<br>9s: p > 0.99
PPX effects on side omission (Ctrl)	Fig. S2E (left)	Within animal	2way ANOVA, Bonferroni	N = 16	NA	PPX: F(1, 60) = 0.03, p = 0.8696, ηp² = 0.0002<br>delay: F(3, 60) = 1.96, p = 0.1303, ηp² = 0.0554<br>delays x PPX: F(3, 60) = 0.77, p = 0.5134, ηp² = 0.0141<br><br>0s: p > 0.99<br>3s: p = 0.99<br>6s: p > 0.99<br>9s: p > 0.99
PPX effects on side omission (Park)	Fig.S2E (right)	Within animal	2way ANOVA, Bonferroni	N = 31	NA	PPX: *F*(1, 120) = 2.06, *p* = 0.1536, ηp² = 0.0035<br>delay: F(3, 120) = 4.98, p = 0.0027, ηp² = 0.0876<br>delays x PPX: F(3, 120) = 0.47, p = 0.7027, ηp² = 0.0024<br><br>0s: p > 0.99<br>3s: p > 0.99<br>6s: p > 0.99<br>9s: p = 0.73
PPX effects on latency to trigger (Ctrl)	Fig. S2F	Within animal	2way ANOVA, Bonferroni	N = 16	NA	Reward size: F(1, 55) = 12.10, p = 0.0010<br>delay: F(3, 60) = 0.4260, p = 0.7351<br>delay x size:

						F(3, 55) = 2.909, p = 0.0426<br><br>0s: p = 0.0019<br>3s: p = 0.087<br>6s: p > 0.99<br>9s: p > 0.99
PPX effects on latency to trigger (Park)	Fig.S2G	Within animal	2way ANOVA, Bonferroni	N = 31	NA	Reward size: F(1, 114) = 7.016, p = 0.0092<br>delay: F(3, 120) = 0.3726, p = 0.7729<br>delay x size: F(3, 114) = 4.838, p = 0.0033<br><br>0s: p = 0.017<br>3s: p = 0.004<br>6s: p >0.99<br>9s: p > 0.99
PPX effects on latency to trigger (Park/PPX)	Fig.S2H	Within animal	2way ANOVA, Bonferroni	N = 31	NA	Reward size: F(1, 117) = 0.1514, p = 0.6979<br>delay: F(3, 120) = 3.021, p = 0.0325<br>delay x size: F(3, 117) = 1.440, p = 0.2347<br><br>0s: p > 0.99<br>3s: p = 0.89<br>6s: p > 0.99<br>9s: p = 0.63
PPX effects on latency to trigger immediate/small rewards (Park)	Fig. S2G, H	Within animal	2way ANOVA, Bonferroni	N = 31	NA	delay: F(3, 118) = 0.667, p = 0.574<br>PPX: F(1, 114) = 4.505, p = 0.020<br>delay x PPX: F(3, 114) = 1.737, p = 0.163<br>0s: p = 0.05<br>3s: p = 0.02<br>6s: p = 0.98<br>9s: p = 0.90
A value during PPX off (Park)	Fig. S2I	Within animal	one-way ANOVA, Bonferroni	N = 31	NA	Park vs. PPX: p < 0.0001<br>PPX vs. PPX off: p < 0.0001
K value during PPX off (Park)	Fig. S2J	Within animal	one-way ANOVA, Bonferroni	N = 31	NA	Park vs. PPX: p = 0.03<br>PPX vs. PPX off: p > 0.99
AUC value during PPX off (Park)	Fig. S2K	Within animal	one-way ANOVA, Bonferroni	N = 31	NA	Park vs. PPX: p < 0.0001<br>PPX vs. PPX off: p = 0.0019

A77636 effects on velocity	Fig. 2B&C	Within animal	WSR	Ctrl:<br>N = 10<br>Park:<br>N = 16	NA	Ctrl:<br>Pre vs. A77636<br>$p = 0.16$<br>Park:<br>Pre vs. A77636<br>$p = 0.02$
DD curve during baseline (Ctrl vs. Park)	Fig. 2D&E	Between group	2way ANOVA, Bonferroni	Ctrl:<br>N = 10<br>Park:<br>N = 16	NA	Depletion: $F(1, 96) = 2.26$, $p = 0.1360$, $\eta p^2 = 0.0159$<br>Delay: $F(3, 96) = 13.59$, $p < 0.0001$, $\eta p^2 = 0.2868$<br>delays x Depletion: $F(3, 96) = 0.07$, $p = 0.9781$, $\eta p^2 = 0.0014$<br><br>0s: $p > 0.99$<br>3s: $p > 0.99$<br>6s: $p > 0.99$<br>9s: $p > 0.99$
A77636 effects on DD curve (Ctrl)	Fig. 2D	Within animal	2way ANOVA, Bonferroni	N = 10	NA	A77636: $F(1, 36) = 1.05$, $p = 0.3115$, $\eta p^2 = 0.0134$<br>delay: $F(3, 36) = 5.50$, $p = 0.0032$, $\eta p^2 = 0.2988$<br>delays x A77636: $F(3, 36) = 0.75$, $p = 0.5315$, $\eta p^2 = 0.0028$<br><br>0s: $p > 0.99$<br>3s: $p = 0.81$<br>6s: $p > 0.99$<br>9s: $p > 0.99$
A77636 effects on DD curve (Park)	Fig. 2E	Within animal	2way ANOVA, Bonferroni	N = 16	NA	A77636: $F(1, 60) = 45.68$, $p < 0.0001$, $\eta p^2 = 0.0495$<br>delay: $F(3, 60) = 12.93$, $p < 0.0001$, $\eta p^2 = 0.3454$<br>delays x A77636: $F(3, 60) = 1.77$, $p = 0.1621$, $\eta p^2 = 0.0058$<br><br>0s: $p = 0.57$<br>3s: $p = 0.0047$<br>6s: $p = 0.0003$<br>9s: $p = 0.0002$
A77636 effects on *A* value (Park)	Fig. 2F	Within animal	Friedman, Dunn's	N = 16	NA	Park vs. A77636:<br>$p = 0.31$

A77636 effects on *K* value (Park)	Fig. 2G	Within animal	Friedman, Dunn's	N = 16	NA	Park vs. A77636: $p = 0.0053$
A77636 effects on AUC value (Park)	Fig. 2H	Within animal	Friedman, Dunn's	N = 16	NA	Park vs. A77636: $p = 0.0008$
A77636 effects on center omission (Ctrl)	Fig. S3A (left)	Within animal	2way ANOVA, Bonferroni	N = 10	NA	A77636: $F(1, 36) = 6.26$, $p = 0.0170$, $\eta p^2 = 0.0349$<br>delay: $F(3, 36) = 6.60$, $p = 0.0011$, $\eta p^2 = 0.2681$<br>delays x A77636: $F(3, 36) = 0.50$, $p = 0.6848$, $\eta p^2 = 0.0084$<br><br>0s: $p > 0.99$<br>3s: $p > 0.99$<br>6s: $p = 0.73$<br>9s: $p > 0.14$
A77636 effects on center omission (Park)	Fig. S3A (right)	Within animal	2way ANOVA, Bonferroni	N = 16	NA	A77636: $F(1, 60) = 1.11$, $p = 0.2971$, $\eta p^2 = 0.0056$<br>delay: $F(3, 60) = 7.32$, $p = 0.0003$, $\eta p^2 = 0.1829$<br>delays x A77636: $F(3, 60) = 0.33$, $p = 0.8040$, $\eta p^2 = 0.0050$<br><br>0s: $p > 0.99$<br>3s: $p > 0.99$<br>6s: $p > 0.99$<br>9s: $p > 0.99$
A77636 effects on side omission (Ctrl)	Fig. S3B (left)	Within animal	2way ANOVA, Bonferroni	N = 10	NA	A77636: $F(1, 60) = 1.27$, $p = 0.2648$, $\eta p^2 = 0.0051$<br>delay: $F(3, 60) = 4.82$, $p = 0.0045$, $\eta p^2 = 0.1455$<br>delays x A77636: $F(3, 60) = 0.51$, $p = 0.6802$, $\eta p^2 = 0.0060$<br>0s: $p > 0.99$<br>3s: $p > 0.99$<br>6s: $p > 0.99$<br>9s: $p = 0.77$
A77636 effects on side omission(Park)	Fig.S3D (right)	Within animal	2way ANOVA, Bonferroni	N = 16	NA	A77636: $F(1, 36) = 0.05$, $p = 0.8268$, $\eta p^2 = 0.0004$,<br>delay: $F(3, 36) = 1.59$, $p = 0.2079$, $\eta p^2 = 0.0804$

						delay x A77636: F(3, 36) = 0.15, p = 0.9277, ηp² = 0.0039<br><br>0s: p > 0.99<br>3s: p > 0.99<br>6s: p > 0.99<br>9s: p > 0.99
A77636 effects on latency to trigger (Ctrl)	Fig.S3C	Within animal	2way ANOVA, Bonferroni	N = 10	NA	Reward size: F(1, 36) = 9.45, p = 0.0040, ηp² = 0.0881<br>delay: F(3, 36) = 0.57, p = 0.6389, ηp² = 0.0236<br>delay x size: F(3, 36) = 1.99, p = 0.1332, ηp² = 0.0556<br><br>0s: p = 0.17<br>3s: p = 0.78<br>6s: p = 0.02<br>9s: p > 0.99
A77636 effects on latency to trigger (Park)	Fig.S3D	Within animal	2way ANOVA, Bonferroni	N = 16	NA	Reward size: F (1, 52) = 11.70, p = 0.0012,<br>delay: F (3, 60) = 0.652, p = 0.58<br>delay x size: F (3, 52) = 0.738, p = 0.54<br><br>0s: p = 0.75<br>3s: p = 0.03<br>6s: p = 0.20<br>9s: p > 0.99
A77636 effects on latency to trigger (Park/A77636)	Fig. S3E	Within animal	2way ANOVA, Bonferroni	N = 10	NA	Reward size: F (1, 55) = 3.463, p = 0.07<br>delay: F (3, 60) = 1.109, p = 0.35<br>delay x size: F (3, 55) = 2.190, p = 0.10<br><br>0s: p = 0.43<br>3s: p > 0.05<br>6s: p > 0.99<br>9s: p > 0.99
A value during A77636 off (Park)	Fig. S3F	Within animal	Friedman, Dunn's	N = 16	NA	Park vs. A77636: p = 0.31

						A77636 vs. A77636 off: p = 0.43
K value during A77636 off (Park)	Fig. S3G	Within animal	Friedman, Dunn's	N = 16	NA	Park vs. A77636: p = 0.0053 A77636 vs. A77636 off: p = 0.96
AUC value during A77636 off (Park)	Fig. S3H	Within animal	Friedman, Dunn's	N = 16	NA	Park vs. A77636: p = 0.0008 A77636 vs. A77636 off: p = 0.43
PPX: *A* and TH^+ DLS	Fig. 3A, left	NA	Pearson's	N = 28	NA	r = 0.30 p = 0.11 r^2 = 0.10
PPX: *A* and TH^+ DMS	Fig. 3A, middle	NA	Pearson's	N = 28	NA	r = 0.51 p = 0.006 r^2 = 0.26
PPX: *A* and TH^+ VS	Fig. 3A, right	NA	Pearson's	N = 28	NA	r = 0.35 p = 0.07 r^2 = 0.12
PPX: *K* and TH^+ DLS	Fig. 3B, left	NA	Pearson's	N = 28	NA	r = 0.34 p = 0.07 r^2 = 0.12
PPX: *K* and TH^+ DMS	Fig. 3B, middle	NA	Pearson's	N = 28	NA	r = -0.02 p = 0.92 r^2 = 0.001
PPX: *K* and TH^+ VS	Fig. 3B, right	NA	Pearson's	N = 28	NA	r = 0.18 p = 0.36 r^2 = 0.03
A77636: *A* and TH^+ DLS	Fig.S4A, left	NA	Pearson's	N = 28	NA	r = -0.11 p = 0.67 r^2 = 0.01
A77636: *A* and TH^+ DMS	Fig.S4A, middle	NA	Pearson's	N = 28	NA	r = -0.28 p = 0.29 r^2 = 0.08
A77636: *A* and TH^+ VS	Fig.S4A, right	NA	Pearson's	N = 28	NA	r = -0.22 p = 0.40 r^2 = 0.05
A77636: *K* and TH^+ DLS	Fig.S4B, left	NA	Pearson's	N = 28	NA	r = -0.06 p = 0.81 r^2 = 0.004
A77636: *K* and TH^+ DMS	Fig.S4B, middle	NA	Pearson's	N = 28	NA	r = -0.22 p = 0.41 r^2 = 0.05
A77636: *K* and TH^+ VS	Fig.S4B, right	NA	Pearson's	N = 28	NA	r = -0.31 p = 0.23 r^2 = 0.09
dMSNs FR: Ctrl vs. Park	Fig. 4B	Between group	MWU	Ctrl: N = 3 Park: N = 5	Ctrl: n = 12 Park: n = 24	p = 0.17

iMSNs FR: Ctrl vs. Park	Fig. 4C	Between group	MWU	Ctrl: N = 4<br>Park: N = 4	Ctrl: n = 10<br>Park: n = 16	$p = 0.70$
PPX effects on dMSNs (Ctrl)	Fig. 4D	Within unit	WSR	N = 3	n = 12	$p = 0.91$
PPX effects on iMSNs (Ctrl)	Fig. 4E	Within unit	WSR	N = 4	n = 10	$p = 0.38$
PPX effects on dMSNs (Park)	Fig. 4F	Within unit	WSR	N = 5	n = 24	$p = 0.01$
PPX effects on iMSNs (Park)	Fig. 4G	Within unit	WSR	N = 4	n = 16	$p < 0.001$
PPX effects on response type (Ctrl vs. Park)	Fig. 4D&F, insets	Between group	Fisher's exact	Ctrl: N = 3<br>Park: N = 5	Ctrl: n = 12<br>Park: n = 24	$p = 0.01$
PPX effects on response type (Ctrl vs. Park)	Fig. 4E&G, insets	Between group	Fisher's exact	Ctrl: N = 4<br>Park: N = 4	Ctrl: n = 10<br>Park: n = 16	$p = 0.002$
PPX effects on response type (Ctrl vs. Park)	Fig. S5D	Between group	Fisher's exact	Ctrl: N = 7<br>Park: N = 11	Ctrl: n = 117<br>Park: n = 214	$p = 0.0003$
CNO effects on DD curve (mCherry)	Fig. 5G	Within animal	2way ANOVA, Bonferroni	N = 15	NA	CNO: $F(1, 56) = 0.97$, $p = 0.3297$, $\eta p^2 = 0.0008$<br>delay: $F(3, 56) = 6.75$, $p = 0.0006$, $\eta p^2 = 0.2536$<br>delays x CNO: $F(3, 56) = 0.15$, $p = 0.9320$, $\eta p^2 = 0.0003$<br><br>0s: $p > 0.99$<br>3s: $p > 0.99$<br>6s: $p > 0.99$<br>9s: $p > 0.99$
CNO effects on DD curve (hM4Di)	Fig. 5H	Within animal	2way ANOVA, Bonferroni	N = 12	NA	CNO: $F(1, 44) = 22.22$, $p < 0.0001$, $\eta p^2 = 0.0536$<br>delay: $F(3, 44) = 10.47$, $p < 0.0001$, $\eta p^2 = 0.3474$<br>delays x CNO: $F(3, 44) = 0.87$, $p = 0.4615$, $\eta p^2 = 0.0063$<br><br>0s: $p > 0.99$<br>3s: $p < 0.01$

						6s: $p = 0.049$<br>9s: $p = 0.027$
CNO effects on DD curve (hM3Dq)	Fig. 5I	Within animal	2way ANOVA, Bonferroni	N = 12	NA	CNO: $F(1, 44) = 42.30$, $p < 0.0001$, $\eta p^2 = 0.0569$<br>delay: $F(3, 44) = 19.46$, $p < 0.0001$, $\eta p^2 = 0.5006$<br>delays x CNO: $F(3, 44) = 1.46$, $p = 0.2388$, $\eta p^2 = 0.0059$<br><br>0s: $p = 0.29$<br>3s: $p = 0.0002$<br>6s: $p = 0.0008$<br>9s: $p = 0.04$
CNO effects on *A* (mCherry)	Fig. 5J (left)	Within animal	WSR	N = 15	NA	$p = 0.89$
CNO effects on *A* (hM4Di)	Fig. 5K (left)	Within animal	WSR	N = 15	NA	$P = 0.11$
CNO effects on *A* (hM3Dq)	Fig. 5L (left)	Within animal	WSR	N = 15	NA	$P = 0.01$
CNO effects on *K* value (mCherry)	Fig. 5J (middle)	Within animal	WSR	N = 15	NA	$p = 0.45$
CNO effects on *K* value (hM4Di)	Fig. 5K (middle)	Within animal	WSR	N = 12	NA	$p = 0.02$
CNO effects on *K* value (hM3Dq)	Fig. 5L (middle)	Within animal	WSR	N = 12	NA	$p < 0.01$
CNO effects on AUC (mCherry)	Fig. 5J (right)	Within animal	WSR	N = 15	NA	$p = 0.56$
CNO effects on AUC (hM3Dq)	Fig. 5K (right)	Within animal	WSR	N = 12	NA	$p < 0.001$
CNO effects on AUC (hM4Di)	Fig. 5L (right)	Within animal	WSR	N = 12	NA	$p < 0.001$
Inhibitory DREADD on oIPSCs	Fig.S6C	Within cell	WSR	N = 2	n = 6	$p < 0.01$
Excitatory DREADD on input-output curve	Fig. S6F	Within cell	2way ANOVA, Bonferroni	N = 3	n = 9	CNO: $F(1, 32) = 17.64$, $p = 0.0002$, $\eta p^2 = 0.0207$<br>Current: $F(3, 32) = 15.17$, $p < 0.0001$, $\eta p^2 = 0.5485$

						current x CNO: $F(3, 32) = 2.18$, $p = 0.1100$, $\eta p^2 = 0.0076$
Excitatory DREADD on firing rate	Fig.S6G	Within cell	WSR	N = 3	n = 9	p = 0.01
Chronic effects of PPX on DD curve (Ctrl)	Fig. 6B	Within animal	2way ANOVA, Bonferroni	N = 16	NA	Park vs. 1st, p > 0.99<br>Park vs. 4th, p > 0.99<br>1st vs. 4th, p > 0.99
Chronic effects of PPX on DD curve (Park)	Fig. 6C	Within animal	2way ANOVA, Bonferroni	N = 31	NA	Park vs. 1st, p < 0.0001<br>Park vs. 4th, p < 0.0001<br>1st vs. 4th, p < 0.0081
Chronic effects of A77636 on DD curve (Ctrl)	Fig. 6D	Within animal	2way ANOVA, Bonferroni	N = 10	NA	Park vs. 1st, p > 0.99<br>Park vs. 4th, p = 0.18<br>1st vs. 4th, p = 0.49
Chronic effects of A77636 on DD curve (Park)	Fig. 6E	Within animal	2way ANOVA, Bonferroni	N = 16	NA	Park vs. 1st, p < 0.0001<br>Park vs. 4th, p < 0.0001<br>1st vs. 4th, p = 0.59
PPX effects on response type 1st vs. 4th (Ctrl)	Fig. 6F	Between group	Fisher's exact	1st: N = 7<br>4th: N = 3	1st: n= 80<br>4th: n= 27	p = 0.11
PPX effects on response type 1st vs. 4th (Park)	Fig. 6g	Between group	Fisher's exact	1st: N = 10<br>4th: N = 8	1st:n=105<br>4th:n = 75	p = 0.002
Chronic effects of PPX on 'increase' (1st vs. 4th)	Fig. 6H	Between group	MWU	1st: N = 6,<br>4th: N = 7	1st: n= 19<br>4th: = 32	p = 0.02
Chronic effects of PPX on 'decrease' (1st vs. 4th)	Fig. 6I	Between group	MWU	1st: N = 9<br>4th: N = 5	1st: n= 34<br>4th: n= 15	p = 0.89
